# Energy Transfer and Radical-Pair Dynamics in Photosystem I with Different Red Chlorophyll *a* Pigments

**DOI:** 10.3390/ijms25074125

**Published:** 2024-04-08

**Authors:** Ivo H. M. van Stokkum, Marc G. Müller, Alfred R. Holzwarth

**Affiliations:** 1 Department of Physics and Astronomy and LaserLaB, Faculty of Science, Vrije Universiteit Amsterdam, De Boelelaan 1081, 1081 HV Amsterdam, The Netherlands; alfred.holzwarth@cec.mpg.de; 2 Max-Planck-Institut für Chemische Energiekonversion, D-45470 Mülheim a.d. Ruhr, Germany; marc.mueller@cec.mpg.de

**Keywords:** photosynthesis, target analysis, transient absorption, ultrafast spectroscopy

## Abstract

We establish a general kinetic scheme for the energy transfer and radical-pair dynamics in photosystem I (PSI) of *Chlamydomonas reinhardtii*, *Synechocystis* PCC6803, *Thermosynechococcus elongatus* and *Spirulina platensis* grown under white-light conditions. With the help of simultaneous target analysis of transient-absorption data sets measured with two selective excitations, we resolved the spectral and kinetic properties of the different species present in PSI. WL-PSI can be described as a Bulk Chl *a* in equilibrium with a higher-energy Chl *a,* one or two Red Chl *a* and a reaction-center compartment (WL-RC). Three radical pairs (RPs) have been resolved with very similar properties in the four model organisms. The charge separation is virtually irreversible with a rate of ≈900 ns^−1^. The second rate, of RP1 → RP2, ranges from 70–90 ns^−1^ and the third rate, of RP2 → RP3, is ≈30 ns^−1^. Since RP1 and the Red Chl *a* are simultaneously present, resolving the RP1 properties is challenging. In *Chlamydomonas reinhardtii*, the excited WL-RC and Bulk Chl *a* compartments equilibrate with a lifetime of ≈0.28 ps, whereas the Red and the Bulk Chl *a* compartments equilibrate with a lifetime of ≈2.65 ps. We present a description of the thermodynamic properties of the model organisms at room temperature.

## 1. Introduction

**Figure 1:**
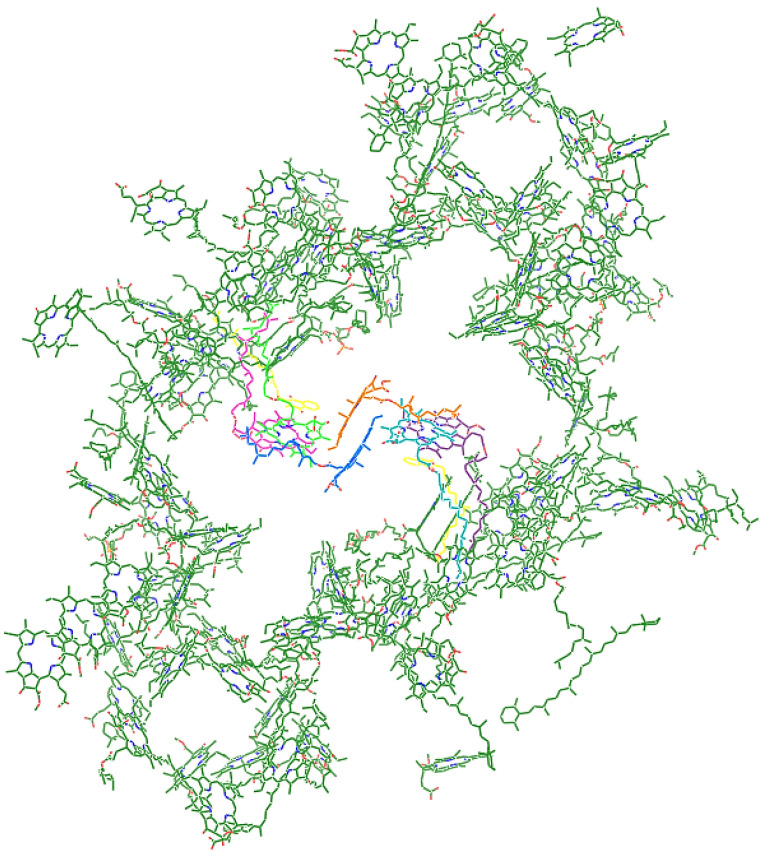
Top view (perpendicular to the periplasmic membrane) of the 96 Chl *a*’s of the PSI complex. The electron-transfer cofactors of the reaction center are shown in different colors: ec1A/ec2B/ec3A, blue/green/magenta; ec1B/ec2A/ec3B, orange/turquoise/purple; PhQA and PhQB, yellow. The structure is based on the *Thermosynechococcus elongatus* PSI structure (PDB ID: 1JB0) [1].

A typical photosystem I (PSI) complex consists of eight proteins that bind more than 90 chlorophyll (Chl) *a* pigments and 22 β-carotenes [1,2] that harvest sunlight and transfer the energy to the reaction center (RC). The charge separation in the PSI RC [1,3,4,5,6] is part of an electron transport chain, the *Z*-scheme of oxygenic photosynthesis [7]. On the lumenal side of the PSI complex, the PSI RC consists of six excitonically coupled Chl *a* pigments [8,9,10,11]. The electron transport chain has two branches (A and B) composed of several cofactors: (i) a Chl *a*’/Chl *a* pair (ec1A/ec1B; orange and blue in the center of Figure 1), traditionally called P700; (ii) a pair of Chl *a* molecules (ec2B/ec3A or ec2A/ec3B) and a phylloquinone (PhQA or PhQB; yellow in Figure 1) in each branch; (iii) an F_X_ iron–sulfur (FeS) cluster where the branches join again, whereby two more FeS clusters (A and B) finalize the delivery of the electron on the stromal side of the membrane, where it is transferred to ferredoxin. Since the six RC Chl *a*’s and a large part of the antenna Chl *a* are bound to the two large subunits (psaA and psaB) that constitute the central core of the PSI complex, the PSI RC cannot be separated from its antenna, and therefore the PSI complex must be studied in its entirety.

Different types of Chl *a*’s that absorb at energies lower than the Bulk Chl *a*, the so-called Red Chl *a*, allow the PSI grown under white light (WL) conditions to utilize light of wavelengths up to 750 nm [12]. To study the mechanism of the charge separation and the radical-pair dynamics experimentally, challenging low-power transient-absorption experiments are mandatory [13,14,15]. Because the excited states of the Red Chl *a* are simultaneously present with the radical pairs of the PSI RC, the interpretation of the measurements is very complicated. Global and target analyses of all measurements using two selective excitations employ a functional compartmental model [16] with microscopic rate constants that connect the different compartments to estimate the parameters that describe the dynamics of the PSI complex [16,17]. Recently, we have established that the PSI complex of *Synechocystis* PCC6803 can thus be described by five excited-state compartments: a Bulk Chl *a* in equilibrium with a higher energy Chl *a,* two Red Chl *a*’s and a reaction center (WL-RC), which consists of six excitonically coupled Chl *a* pigments [18]. The charge separation was described by a simplified scheme with two radical pairs, RP1 and RP2. Here we set out to also study WL-PSI complexes of the well-known model organisms *Chlamydomonas reinhardtii, Thermosynechococcus elongatus* and *Spirulina platensis.* It will be demonstrated that modified versions of the kinetic scheme that describes the PSI complex of *Synechocystis* PCC6803 are needed to describe these new transient-absorption experiments. The modifications are in regard to the properties of the Red Chl *a* compartments and to the microscopic rate constants, suggesting that the WL-RC and radical-pair dynamics are very similar in these four model organisms.

## 2. Results 

### 2.1. Absorption Spectra

**Figure 2:**
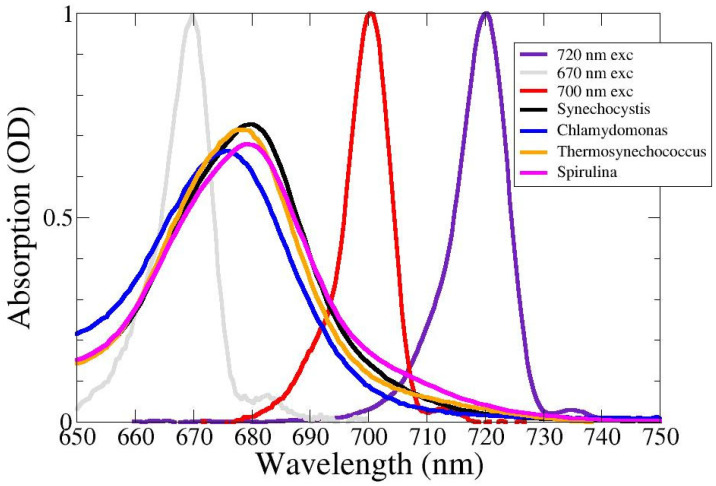
Absorption spectra of WL-PSI complexes of *Chlamydomonas reinhardtii* (blue), *Synechocystis* PCC6803 (black), *Thermosynechococcus elongatus* (orange) and *Spirulina platensis* (magenta) and spectra of the excitatory pulses. Key: 670 (gray), 700 (red) and 720 nm (purple).

The absorption spectra of the WL-PSI complexes are depicted in Figure 2, together with the spectra of the excitatory pulses. The Q_y_ absorption of *Chlamydomonas reinhardtii* (blue in Figure 2) is blue-shifted relative to that of the three cyanobacteria. The absorption of *Spirulina platensis* (magenta) extends up to ≈740 nm, that of *Thermosynechococcus elongatus* (orange) up to ≈730 nm and that of *Synechocystis* PCC6803 (black) up to ≈725 nm. This is consistent with the 6K absorption spectra reported in [12].

### 2.2. Chlamydomonas reinhardtii

**Figure 3:**
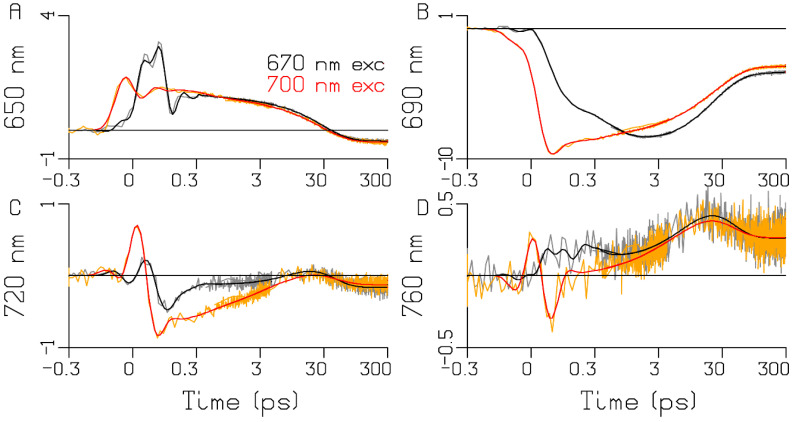
Transient-absorption data and global-analysis fit of WL-PSI complexes of *Chlamydomonas reinhardtii.* Transient absorption (in units of mOD) of *Chlamydomonas reinhardtii* WL-PSI is shown in (**A**–**D**) for four selected wavelengths (indicated in the ordinate label). Key: 670 nm excitation (gray); 700 nm excitation (orange). Black and red lines indicate the target-analysis fit. Note that the time axis is linear until 0.3 ps and logarithmic thereafter. Note also that each panel is scaled to its maximum. The overall rms error of the fit was 0.056 mOD.

**Figure 4:**
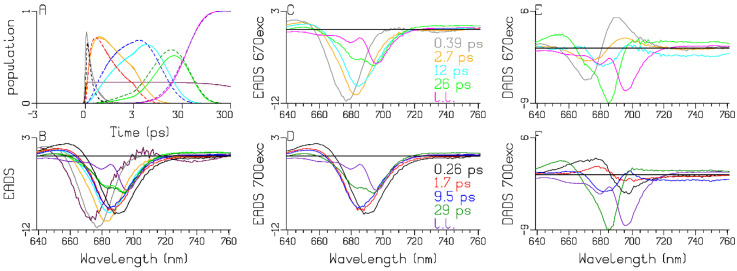
Populations (**A**) of the sequential schemes. Line-type key: 670 nm excitation (solid), 700 nm excitation (dashed). Color key for 670 excitation: gray, 0.39 ps; orange, 2.7 ps; cyan, 12 ps; green, 26 ps; magenta, long lived. Color key for 700 excitation: black, 0.26 ps; red, 1.7 ps; blue, 9.5 ps; dark green, 29 ps; purple, long lived. Maroon in (**A**,**B**) represents the LHCI contribution that decays with a lifetime of 1.4 ns. EADS (in mOD) of the PSI complexes of *Chlamydomonas reinhardtii* estimated with 670 (**C**) and 700 (**D**) nm excitation, and their overlay (**B**). DADS (in mOD) (**E**,**F**).

**Figure 5:**
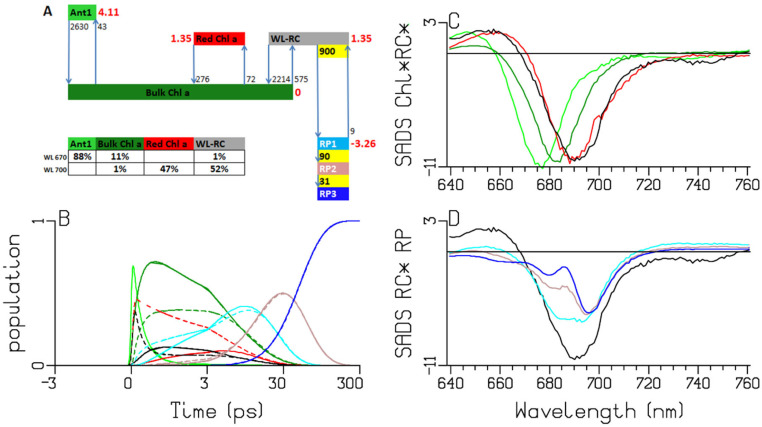
Target analysis of the transient absorption from WL-PSI complexes of *Chlamydomonas reinhardtii.* (**A**) Kinetic scheme with rates in ns^−1^. Each compartment is represented by a colored box. The initial populations (in %) are indicated in the excited-state boxes for the 670 or 700 nm excitation. Red numbers indicate the Gibbs free energy (in k_B_T = 25.2 meV, 20 °C) relative to Bulk Chl *a*. For clarity, the natural decay rates of the excited states and the LHCI have been omitted. Populations (**B**) and SADS (**C**,**D**, in mOD). Line-type key: 670 nm excitation (solid), 700 nm excitation (dashed). Color key: Ant1 (light green), Bulk Chl *a* (dark green), Red Chl *a* (red), WL-RC (black), RP1 (cyan), RP2 (brown) and RP3 (blue). Note that the time axis in (**B**) is linear until 3 ps and logarithmic thereafter.

The PSI complex of the well-known model organism *Chlamydomonas reinhardtii* has been widely studied [15,19,20,21,22]. However, in studies of its charge separation, the presence of Red Chl *a* in the core has not yet been considered. In the ultrafast transient-absorption experiments on WL-PSI complexes of *Chlamydomonas reinhardtii* in the reduced form, two excitation wavelengths have been used: 670 nm, which selectively excites Chl *a* pigments absorbing to the blue of the Bulk Chl *a* [16,18,23,24]; and 700 nm, which excites the RC and the Red Chl *a* pigments (Figure 2). Representative traces and fits are depicted in Figure 3. The quality of the fit is excellent (Figure 3, Figures S2 and S3). The level near 300 ps can be attributed to the difference in absorption of the final radical pair. A small amount of uncoupled light-harvesting complex I (LHCI) contamination is responsible for the slight difference in the final level of the traces. Here, we display four traces where the LHCI contribution is very small (cf. the LHCI SADS; maroon in Figure 4). The 760 nm trace (Figure 3D) demonstrates the rise of the anion absorption, which decays to a lower final level at ≈30 ps. The difference between the colored traces representing the two excitation wavelengths is very informative. The kinetics for the 670 (black) and 700 (red) nm excitations differ until ≈10 ps. A small amount of relatively slow equilibration is clearly visible near 720 nm (Figure 3C, where the black and red lines cross near 10 ps). This must be attributed to the Red Chl *a* pigments that have been selectively excited with 700 nm and that equilibrate slowly with the Bulk Chl *a* antenna. A “coherent artefact” (CA) straddling time zero is present in the transient-absorption data in Figure 3. The analysis thereof is presented and discussed in Figure S1 and below in Section 3.1. First, we present the global-analysis results of each experiment using a sequential kinetic scheme with five components with increasing lifetimes. With 670 nm excitation, an additional LHCI component is used. This is the minimal number of components needed for a satisfactory fit (Figure 3, Figures S2 and S3). The populations of the sequential schemes are depicted in Figure 4A. The evolution-associated difference spectra (EADS) estimated with 670 and 700 nm excitations are depicted in Figure 4C,D, respectively, whereas the accompanying decay-associated difference spectra (DADS) are shown in Figure 4E,F. With the 670 nm excitation, the gray to orange evolution (0.39 ps) can be attributed to ultrafast equilibration of the initially excited antenna states with the Bulk Chl *a* states. The orange to cyan evolution (2.7 ps) can be attributed to equilibration between the Bulk Chl *a* and the Red Chl *a* state. The green EADS (evolving in 12 ps) is dominated by features of the first radical pair, whereas the magenta EADS (evolving in 26 ps) can be attributed to the final radical pair. With the 700 nm excitation (Figure 4B), the first lifetime has been estimated as 0.26 ps (black EADS; Figure 4D) and is a mixture of the initially excited states (attributable to the excited Red Chl *a* and WL-RC). Importantly, its DADS (black in Figure 4F) is conservative, with a loss of bleach plus stimulated emission (BL+SE) at around 700 nm and a gain at around 680 nm. This is attributed to ultrafast equilibration between the excited WL-RC and the Bulk Chl *a* antenna. The 1.7 and 9.5 ps DADS (red and blue in Figure 4F) are complex, and the 29 ps DADS (dark green) is dominated by features of the first radical pair. The long-lived purple EADS can be attributed to the final radical pair; it is identical for the two excitation wavelengths. These eleven EADS and DADS can only be interpreted with the help of a target analysis using the kinetic scheme of Figure 5A.

To characterize the trapping, the data are simultaneously analyzed with a simplified kinetic scheme (Figure 5A) which is a modified version of the scheme from [18]. Briefly, trapping occurs by a WL-RC compartment, which consists of six excitonically coupled Chl *a* pigments. Bulk Chl *a* is in equilibrium with a higher-energy Chl *a* (preferentially excited with 670 nm), one Red Chl *a* and a WL-RC compartment. The mechanism of the charge separation is highly simplified, neglecting the equilibration within the RC and disregarding the charge separation in the two separate branches, A and B (Figure 1) [15,19,21]. It is further assumed that the charge separation can be approximated by a three-step process. We found that the data could not be fitted satisfactorily by a kinetic scheme with fewer compartments. To perform a simultaneous target analysis with a minimal number of species-associated difference spectra (SADS), all SADS have been linked between the two experiments. The estimated trapping is virtually irreversible, with a forward rate of ≈900 ns^−1^ and a recombination rate of ≈9 ns^−1^, in agreement with previous findings [18,25]. The subsequent rates of electron transfer are ≈90 and ≈30 ns^−1^. An important difference with the scheme from [18] is the faster equilibration between the excited WL-RC and the Bulk Chl *a* compartments.

First, we will discuss the populations in Figure 5B, which are computed using the kinetic scheme in Figure 5A. The model for the observations and the computation of the populations are detailed in Appendix A. The populations are described by a sum of exponential decays (convolved with the instrument response function (IRF)) with associated amplitudes that are summarized in an amplitude matrix. The amplitude matrices after 670 and 700 nm excitations are given in Table 1A and Table 1B, respectively. After the 670 nm excitation, ultrafast equilibration takes place with a lifetime of 0.38 ps (light and dark green solid lines). Subsequently, the RC population rises, with a lifetime of 0.28 ps (black solid line). With a lifetime of 2.65 ps, further equilibration between the Bulk Chl *a* and the Red Chl *a* takes place, and the RP1 population rises, with a lifetime of 7.9 ps (cyan solid line). The RP2 population rises with a lifetime of 13 ps (brown solid line), which subsequently decays to RP3 with a lifetime of ≈32 ps (blue solid line). The excitations are trapped from the equilibrated system with a lifetime of ≈13 ps. The main differences with 700 nm excitation are the virtual absence of the 0.37 ps equilibration and the immediate rise of both Bulk Chl *a* and RP1 with a lifetime of 0.28 ps (dark green and cyan dashed line), since ≈52% of the excitations directly excites the RC (black dashed line). In addition, the directly excited Red Chl *a* population persists (red dashed line) until it decays via trapping from the equilibrated system with a lifetime of ≈13 ps. The RP3 quantum yield in PSI is ≈99% (Table 1; “long lived” column). The dynamics of the LHCI contamination, excited only with 670 nm, is detailed in Figure S4.

Next, we discuss the estimated SADS. The Bulk Chl *a* SADS (dark green in Figure 5C) exhibits a broad BL+SE band with a minimum at 682 nm, and a small excited-state absorption (ESA) below 660 nm. With the 670 nm excitation, the initially excited antenna compartment Ant1 (light green) is resolved, with the SADS shifted to a higher energy relative to the Bulk Chl *a* SADS (light vs. dark green in Figure 5C). The estimated SADS of the Red Chl *a* species exhibits a broad BL+SE band with a minimum near 689 nm and an ESA with a maximum at around 660 nm (red in Figure 5C). The WL-RC SADS (black in Figure 5C,D) exhibits a broad BL+SE band with a minimum at around 691 nm and an ESA below 665 nm. Note that the Red Chl *a* SADS is very similar to the WL-RC SADS. The RP3 SADS demonstrates the bleaching of the cation ($P_{700}^{+}$) with a superimposed electrochromic shift at 690 nm (blue in Figure 5D). The phylloquinone anion of RP3 does not absorb in this wavelength region. The SADS of RP1 shows bleaching at around 690 nm and the SADS of RP2 displays a shoulder at around 685 nm, which agree with the literature [15,21,26]. The difference between the RP1/RP2 and the RP3 SADS can be attributed to the Chl *a* anions of RP1/RP2, which absorb below 660 and above 700 nm (compare the cyan and blue SADS in Figure 5D). The size of this difference is smaller than the SADS of the Bulk Chl *a*, since the latter also contains the stimulated emission.

### 2.3. Synechocystis PCC6803

**Figure 6:**
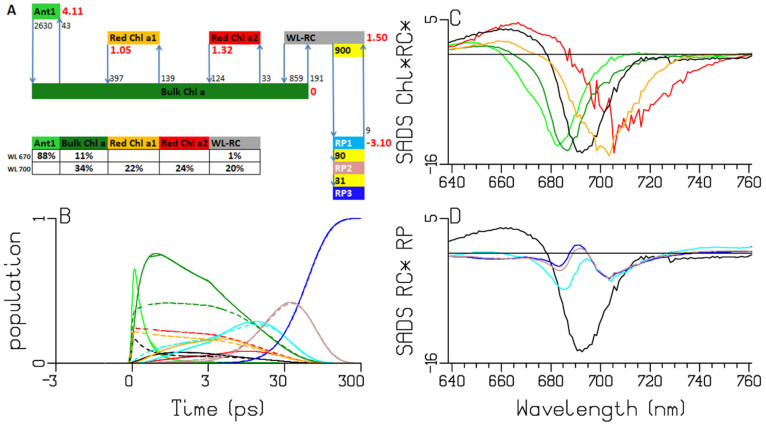
Target analysis of the transient absorption from WL-PSI complexes of *Synechocystis* PCC6803. (**A**) Kinetic scheme with rates in ns^−1^. Each compartment is represented by a colored box. The initial populations (in %) are indicated in the excited-state boxes for 670 or 700 nm excitation. Red numbers indicate the Gibbs free energy (in k_B_T = 25.2 meV, 20 °C) relative to Bulk Chl *a*. For clarity, the natural decay rates of the excited states and free Chl *a* have been omitted. Populations (**B**) and SADS (**C**,**D**, in mOD). Line-type key: 670 nm excitation (solid), 700 nm excitation (dashed). Color key: Ant1 (light green), Bulk Chl *a* (dark green), Red Chl *a1* (orange) and *a2* (red), WL-RC (black), RP1 (cyan), RP2 (brown) and RP3 (blue). Note that the time axis in (**B**) is linear until 3 ps and logarithmic thereafter.

*Synechocystis* PCC6803 absorbs light until 725 nm [12] (Figure 2), which is due to the presence of two Red Chl *a* pools that absorb to the red of the RC. We revisit the *Synechocystis* PCC6803 target analysis from [18] using a kinetic scheme with three instead of two RP compartments. The fit is excellent (Figure S7). Here we mainly discuss the RP dynamics. Note that the rate constants RP1 → RP2 and RP2 → RP3 are virtually identical between *Chlamydomonas reinhardtii* and *Synechocystis* PCC6803. However, the Red Chl *a* compartments are very different, with their SADS red-shifted relative to the WL-RC SADS. The RP3 SADS shows a large electrochromic shift at 688 nm (blue in Figure 6D), which is already present in RP1 and RP2. The RP1 SADS shows a bleaching with a minimum at 685 nm, which decreases in RP2 (cyan and brown lines in Figure 6D).

### 2.4. Thermosynechococcus elongatus

**Figure 7:**
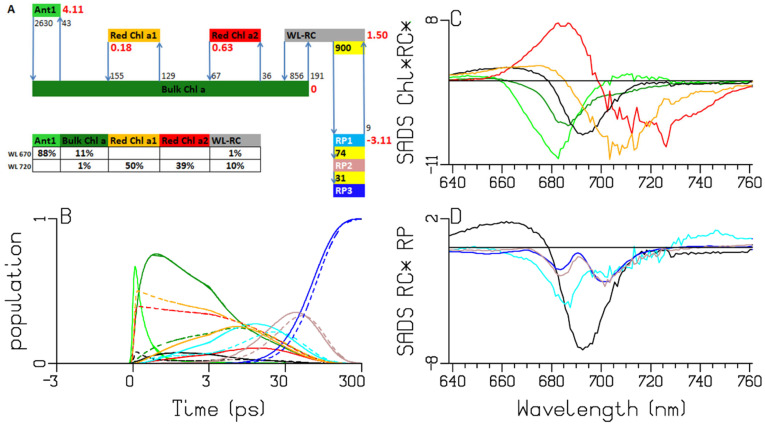
Target analysis of the transient absorption from WL-PSI complexes of *Thermosynechococcus elongatus.* (**A**) Kinetic scheme with rates in ns^−1^. Each compartment is represented by a colored box. The initial populations (in %) are indicated in the excited-state boxes for 670 or 720 nm excitation. Red numbers indicate the Gibbs free energy (in k_B_T = 25.2 meV, 20 °C) relative to Bulk Chl *a*. For clarity, the natural decay rates of the excited states have been omitted. Populations (**B**) and SADS (**C**,**D**, in mOD). Line-type key: 670 nm excitation (solid), 720 nm excitation (dashed). Color key: Ant1 (light green), Bulk Chl *a* (dark green), Red Chl *a1* (orange) and *a2* (red), WL-RC (black), RP1 (cyan), RP2 (brown) and RP3 (blue). Note that the time axis in (**B**) is linear until 3 ps and logarithmic thereafter.

It is well known that *Thermosynechococcus elongatus* absorbs even more to the red than *Synechocystis* PCC6803 [12] (Figure 2). Again, the target-analysis fit is excellent (Figure S10). We discuss the differences between the two target-analysis results in Figure 6 and Figure 7. The Gibbs free-energy differences relative to Bulk Chl *a*, which were 1.05 and 1.32 k_B_T, now decrease to 0.18 and 0.63 k_B_T, which can be explained by the shift to lower energy in the Red Chl *a* pools. The equilibration between the Red Chl *a* pools and the Bulk Chl *a* slows down because of the reduced Förster overlap. The RP3 SADS shows a small electrochromic shift at 690 nm (blue in Figure 7D), which is already present in RP1 and RP2. The RP1 and RP2 SADS show the same trends as in Figure 5D and Figure 6D. The estimated RP1 → RP2 rate constant is smaller: 74 vs. 90 ns^−1^.

### 2.5. Spirulina Platensis

**Figure 8:**
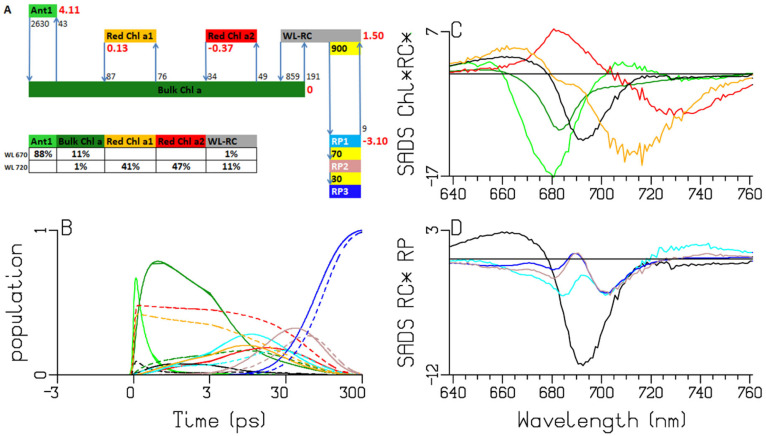
Target analysis of the transient absorption from WL-PSI complexes of *Spirulina platensis*. (**A**) Kinetic scheme with rates in ns^−1^. Each compartment is represented by a colored box. The initial populations (in %) are indicated in the excited-state boxes for 670 or 720 nm excitation. Red numbers indicate the Gibbs free energy (in k_B_T = 25.2 meV, 20 °C) relative to Bulk Chl *a*. For clarity, the natural decay rates of the excited states and the free Chl *a* have been omitted. Populations (**B**) and SADS (**C**,**D**, in mOD). Line-type key: 670 nm excitation (solid), 720 nm excitation (dashed). Color key: Ant1 (light green), Bulk Chl *a* (dark green), Red Chl *a1* (orange) and *a2* (red), WL-RC (black), RP1 (cyan), RP2 (brown) and RP3 (blue). Note that the time axis in (**B**) is linear until 3 ps and logarithmic thereafter.

Trimers of *Spirulina platensis* absorb until 740 nm [12] (Figure 2). The target analysis fit is excellent as well (Figure S13). The Gibbs free-energy differences relative to Bulk Chl *a* decrease to 0.13 and −0.37 k_B_T (Figure 8), which can be explained by the further shift to a lower energy of the BL+SE of the Red Chl *a* pools. Below, we will demonstrate how these Gibbs free-energy differences can be interpreted thermodynamically. After the 720 nm excitation, the population of the red-most Chl *a2* (dashed red lines in Figure 8B) dominates until ≈100 ps, and consequently, the rise of RP is delayed. Its SADS has a BL+SE extremum at ≈732 nm. Since RP1 and both Red Chl *a* populations are simultaneously present, resolving the RP1 properties is the most challenging with this model organism.

**Figure 9:**
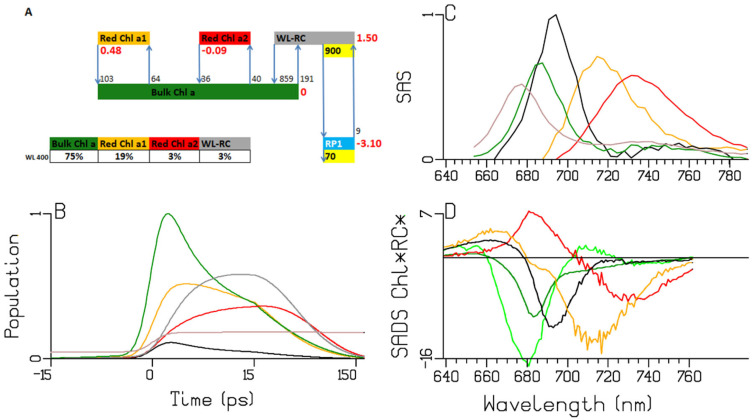
Target analysis of the emission from WL-PSI complexes of *Spirulina platensis*. (**A**) Kinetic scheme with rates in ns^−1^. Each compartment is represented by a colored box. The initial populations (in %) are indicated in the excited-state boxes for the 400 nm excitation. Red numbers indicate the Gibbs free energy (in k_B_T = 25.2 meV, 20 °C) relative to Bulk Chl *a*. For clarity, the natural decay rates of the excited states and the free Chl *a* have been omitted. Populations (**B**), SAS (**C**) and SADS (**D**, in mOD) are copied from Figure 8C. Color key: Bulk Chl *a* (dark green), Red Chl *a1* (orange) and *a2* (red), WL-RC (black), RP1 (cyan) and free Chl *a* (brown). Note that the time axis in (**B**) is linear until 15 ps and logarithmic thereafter.

To test the applicability of the kinetic scheme from Figure 8A, we performed a target analysis of the emission data from [12]. In the target of the emission data, it was assumed that the area of the excitonic species-associated spectra (SAS) (WL-RC and Red Chl *a1* and *a2*) is 35% larger than the Bulk Chl *a* SAS area [16,18]. The target-analysis fit quality (Figure S15) is excellent. The SAS estimated from the emission data (Figure 9B) are consistent with the BL+SE parts of the SADS (cf. Figure 9; panels C and D). Note that in the emission, RP1 has a zero SAS, and Ant1 is not resolvable. The independent estimates of the equilibria between the Red Chl *a* and the Bulk Chl *a* compartments are also consistent (cf. Figure 8A and Figure 9A).

## 3. Discussion

### 3.1. Sub-ps Dynamics

The sub-ps dynamics in the transient-absorption signals from WL-PSI complexes are very complex (cf. the data and fits in Figure 3, Figures S2, S7, S10 and S13). In our model for the observations (Appendix A), these sub-ps signals are described by a superposition of damped oscillations, IRF derivatives, ultrafast equilibration with the Bulk Chl *a* compartment of the Ant1 compartment after a 670 nm excitation and of the WL-RC compartment after a 700 nm excitation. The spectra associated with the damped oscillations and the IRF derivatives are summarized in Figures S1, S6, S9 and S12. With a 670 nm excitation, strong oscillations are present, which can be described by fast damped oscillations of ≈450 cm^−1^. In addition, slowly damped oscillations of ≈20 cm^−1^ were needed with *Thermosynechococcus elongatus* and *Spirulina platensis.* The IRFAS in panels B of Figures S1, S6, S9 and S12 are also very complex. No attempt is made here to interpret these signals, which are a mixture of CAs, four-wave mixing and damped oscillations. After 700 or 720 nm excitation, apart from these CA-related signals, contributions are expected from the ultrafast equilibration within the WL-RC and from the Stokes shift of the Red Chl *a*. An alternative kinetic scheme with a faster rate of primary charge separation has been proposed in [8,27,28,29,30]. We demonstrated in *Chlamydomonas reinhardtii* (Figure 5A and Table 1) that after direct excitation of the WL-RC, the RP1 population rises with a lifetime of 0.28 ps, but this ultrafast lifetime is mainly due to the outward rate of WL-RC → Bulk Chl *a* (≈2200 ns^−1^), which is much larger than the intrinsic rate of charge separation of ≈900 ns^−1^. Structure-based (Figure 1) computations of PSI exciton dynamics show that the RC is well connected to the antenna, with nearest neighbor inter-pigment energy-transfer rates ranging from ≈300–≈11,000 ns^−1^ [31,32,33,34,35,36]. An intrinsic primary charge separation rate of ≈900 ns^−1^ is predicted in [32]. It would be interesting to compare our results to predictions of transient-absorption spectra [31,37].

### 3.2. Thermodynamic Considerations

The estimated SADS of the excited states in Figure 5C, Figure 6C, Figure 7C, Figure 8C exhibit smooth ESA and BL+SE bands. The location of the extremum of the BL+SE band (in wavenumber, ${\bar{\nu }}_{\max}$, or in wavelength, $\lambda_{\max}$) in the SADS can be estimated with the help of skewed Gaussian shapes [17]. The quality of these spectral fits is demonstrated in Figure S5. The estimated ${\bar{\nu }}_{\max}$ is employed to compute the enthalpy difference ΔH relative to Bulk Chl *a* in units of k_B_T. Using the Gibbs free-energy difference (relative to Bulk Chl *a*) ΔG computed from the kinetic schemes in Figure 5A, Figure 6A, Figure 7A and Figure 8A, the entropy difference (relative to Bulk Chl *a*) is computed from ΔH − ΔG = TΔS. Finally, the number of pigments in the compartments, “N”, is estimated from the entropy difference ($N=N_{Bulk}\exp(\Delta S/k_{B})$) and shown in the bottom rows of Table 2A–D, where we have used an estimate of $N_{Bulk}=78$ throughout. This results in estimates of ≈2 pigments for the Ant1 compartment, somewhat less than 2 Red Chl *a2* pigments and 4–10 pigments for the Red Chl *a1* compartment. The estimate for the WL-RC compartment of ≈eight pigments is somewhat larger than the expected six pigments. 

Precise quantification of the number of pigments in each compartment is difficult, since it crucially depends on the validity of the many assumptions that have been used in the target analysis. Also, in view of the excitonic interactions involved in many of the compartments, the values of “N” in the bottom rows of Table 2A–D should be interpreted cautiously. The presence of a large Stokes’ shift of up to 18 nm [12,18] overestimates ΔH in Red Chl *a2* and explains the small “N” values estimated with *Thermosynechococcus elongatus* and *Spirulina platensis.*

Below room temperature, the entropy advantage of the Bulk Chl *a* decreases linearly with the temperature, and therefore the Gibbs free-energy difference (relative to Bulk Chl *a*) of the WL-RC and the Red Chl *a* compartments will become negative. At a cryogenic temperature (77 K), the heterogeneity of the PSI complexes will come into play, and a homogeneous kinetic model will no longer applicable [38].

### 3.3. Comparison of the RC and RP SADS

**Figure 10:**
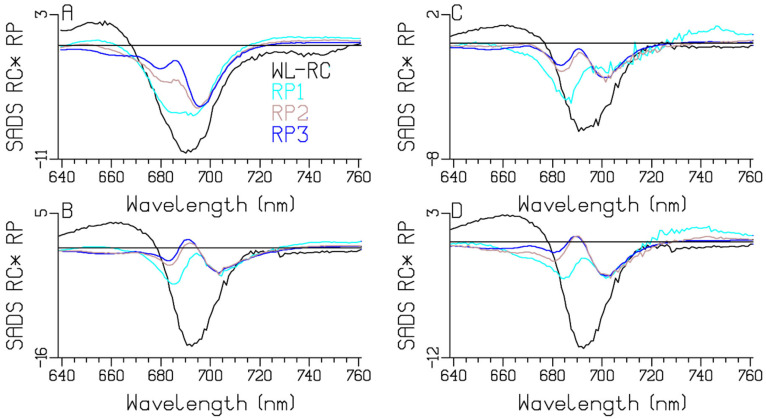
RC and RP SADS (in mOD) estimated from target analysis of the transient absorption from WL-PSI complexes of *Chlamydomonas reinhardtii* (**A**), *Synechocystis* PCC6803 (**B**), *Thermosynechococcus elongatus* (**C**), and *Spirulina platensis* (**D**). Color key: WL-RC (black), RP1 (cyan), RP2 (brown) and RP3 (blue).

The estimated RC and RP SADS are collated in Figure 10. Note the strong resemblances of the trends in the shapes. The shape of the RC SADS (black) shows a BL+SE minimum at around 690 nm in *Chlamydomonas reinhardtii* (Figure 10A) and at around 693 nm in the cyanobacteria, a nice SE above 720 nm, and a nice ESA below 670 or 680 nm. The RP3 SADS (blue) demonstrates the bleaching of the cation ($P_{700}^{+}$), with a superimposed electrochromic shift at around 690 nm that differs among the four model organisms. Recall that the phylloquinone anion of RP3 does not absorb in this wavelength region. The RP1 (cyan) and RP2 (brown) SADS are best judged relative to this RP3 SADS (blue). The SADS of RP1 (cyan) differs most from the RP3 SADS (blue) at around 685 nm, which can be attributed to the bleaching of the Chl *a* anion. Additionally, this anion absorbs below ≈660 nm and above ≈700 nm (compare the cyan and blue SADS). The RP2 SADS (brown) is in-between that of the RP1 (cyan) and RP3 SADS (blue), with less anion bleaching and absorption. A target analysis of transient-absorption data from mutants of *Chlamydomonas reinhardtii* established different charge-separation properties in the A and B branches of the PSI RC [8,14,15,21], which will be revisited, considering the presence of the here-established Red Chl *a*.

## 4. Materials and Methods

### 4.1. Transient-Absorption Experiments

Ultrafast transient-absorption experiments on PSI complexes from *Chlamydomonas reinhardtii* [13,39], *Synechocystis* PCC6803 (hereafter SCy6803) [40], *Thermosynechococcus elongatus* [1,41] and *Spirulina platensis* [42,43] were performed at RT (20 °C) under annihilation-free conditions [13,15,44] with excitation at 670 and 700 or 720 nm (Figure 2). A regenerative titanium–sapphire laser system was employed, which pumped an optical parametric amplifier (OPA) to excite the samples at various excitation wavelengths with an energy of a few hundred picojoules in a ≈125 μm diameter spot at a repetition rate of 3 kHz. The probe white-light continuum was generated as a weak single filament in a thin 1–2 mm sapphire plate. The IRF was described by a Gaussian shape of ≈130 fs full width at half-maximum (FWHM).

For the isolated PSI samples, 10–40 μM phenazine methosulfate (PMS) and 20–50 mM sodium ascorbate were added as described in order to keep the RCs in an open, i.e., reduced, state [13]. The OD of the sample was ≈0.7/mm at 680 nm (Figure 2). Furthermore, it was necessary to use a rotating cuvette, which was periodically shifted in the horizontal plane to ensure a long time to recover, in the order of 1 min. Time-gated spectra from 638.5 to 761.5 nm were measured in two time ranges: from −1 to 4.8 ps, in steps of 13.3 fs, and from 1 to 300 ps, in steps of 0.5 ps.

The excitation intensity was kept below the annihilation threshold. For example, the samples have a high total optical density of 0.7 to 0.8 in the 1 mm path length of the rotating and shifting cuvette. The induced bleaching at time zero is usually below 0.01 ∆OD, which includes also the contribution from the stimulated emission. Thus, the excitation probability is in the order of 0.01/0.7/2 (0.7%). For a PSI complex with 90 Chls, this probability yields an average of μ = 0.6 photons/complex. The Poisson statistic $p(k)=\mu^{k}\exp(-\mu )/k!$ predicts the probability for double excitations (k=2) to be less than 10%.

A small contamination of LHCI was present in *Chlamydomonas reinhardtii.* With SCy6803 and *Spirulina platensis*, a small contamination of free Chl *a* was present. These contaminations only appear in the 670 nm excitation experiments and are described by a long-lived component in the global and target analysis of the time-resolved spectra.

### 4.2. Time-Resolved Fluorescence Spectra

Time-resolved fluorescence spectra from 653 to 789 nm of the PSI complex of *Spirulina platensis* [43] have been measured at RT (20 °C) with a Hamamatsu C5680 synchroscan streak camera combined with a Chromex 250IS spectrograph [12]. The FWHM of the IRF was ≈3.9 ps. The global analysis and a simplified target analysis of the time-resolved fluorescence spectra of *Spirulina platensis* after 400 nm excitation have been presented in [12].

### 4.3. Global and Target Analysis of Time-Resolved Spectra

The global- and target-analysis methodologies have been described in [17,45], and the relevant equations are explained in Appendix A. A Gaussian-shaped IRF of ≈130 fs FWHM is used, with parameters *μ* for the time of the IRF maximum and Δ for the FWHM of the IRF. The wavelength dependence of the parameter μ is described by a first-order polynomial in the wavenumber domain [46]. A CA straddling time zero is present in the transient absorption of Figure 2, Figures S2, S7, S10 and S13. This is modelled with the term IRF(μ,Δ)⋅IRFAS. It contains a matrix, IRF(μ,Δ), with the zeroth, first and second derivatives of the IRF [45,46] (cf. Figure S1A). In addition, damped oscillations [45] are needed to describe the CA and spectral evolution during the first 300 fs (Figures S1, S6, S9 and S12).

The A panels of Figure 5, Figure 6, Figure 7 and Figure 8 summarize the different kinetic schemes for the WL-PSI samples. Among these samples, the common SADS (Bulk Chl *a* (dark green), Red Chl *a1* (orange), Red Chl *a2* (red), WL-RC (gray)) and the rate parameters are linked as much as possible. Resolving the SADS of the Red Chl *a,* WL-RC and RP1 compartments in combination with the equilibria is very hard; it requires some preknowledge in the form of reasonable guidance spectra [47] in order to arrive at consistent and interpretable SADS, and consistent and interpretable thermodynamic properties (cf. Table 2). The relative precision of the estimated parameters is 10%.

## 5. Conclusions

We conclude that our kinetic schemes describe the entire energy transfer and trapping in the WL-PSI complexes of the four model organisms, which mainly differ in the properties of their Red Chl *a* pigments. The charge separation and radical-pair dynamics have much in common (Figure 5, Figure 6, Figure 7, Figure 8 and Figure 10) and demonstrate that a general kinetic scheme can describe the energy transfer and radical-pair dynamics in PSI of *Chlamydomonas reinhardtii* and the cyanobacteria *Synechocystis* PCC6803, *Thermosynechococcus elongatus* and *Spirulina platensis* grown under WL conditions. The shapes of the Red Chl *a* SADS are generally consistent in the four model organisms, with strong ESAs ranging from 670 to 695 nm and a BL+SE minimum ranging from 690 to 730 nm. The target-analysis results are thermodynamically interpretable (Table 2). In the model organism with the least Red Chl *a, Chlamydomonas reinhardtii,* the excited WL-RC and Bulk Chl *a* compartments equilibrate with a lifetime of ≈0.28 ps, whereas the Red and the Bulk Chl *a* compartments equilibrate with a lifetime of ≈2.65 ps. The observed trapping lifetime of the equilibrated antenna and WL-RC is ≈13 ps. However, the charge separation is virtually irreversible with an intrinsic rate of ≈900 ns^−1^. This can thus be considered trap-limited kinetics; see [6] and references cited therein. The results presented here are generally consistent with previous analyses and publications [6,10,12,13,14,15,18,25,48]. However, the simultaneous analysis of the data obtained with the different excitation wavelengths in the different model organisms results in a more consistent and universal view that enhances the general understanding of the energy transfer and the charge separation in WL-PSI.

## Figures and Tables

**Table 1:** Amplitude matrices of the reduced PSI complex of *Chlamydomonas reinhardtii* after 670 (**A**) or 700 (**B**) nm excitation. Key: Ant1 (light green), Bulk Chl *a* (dark green), Red Chl *a* (red), WL-RC (black), RP1 (cyan), RP2 (brown) and RP3 (blue).

**A**	**species\τ (ps)**	0.28	0.38	2.65	7.92	13.3	32.3	long lived	input
	**Ant1**	0.015	0.850	0.007	0.005	0.004	0	0	0.88
	**Bulk Chl *a***	−0.335	−0.475	0.361	0.316	0.241	0	0	0.11
	**Red Chl *a***	0.007	0.014	−0.258	0.150	0.086	0	0	0
	**WL-RC**	0.418	−0.589	0.075	0.055	0.051	0	0	0.01
	**RP1**	−0.108	0.208	−0.242	−1.826	1.968	0	0	0
	**RP2**	0.003	−0.007	0.063	1.724	−3.984	2.201	0	0
	**RP3**	0.000	0.000	−0.005	−0.423	1.637	−2.203	0.994	0
	**sum**	0	0	0	0	0.004	−0.002	0.994	1
									
**B**	**species\τ (ps)**	0.28	0.38	2.65	7.92	13.3	32.3	long lived	input
	**Ant1**	0.016	−0.022	−0.005	0.006	0.004	0	0	0
	**Bulk Chl *a***	−0.355	0.012	−0.273	0.371	0.255	0	0	0.01
	**Red Chl *a***	0.008	0.000	0.195	0.177	0.091	0	0	0.47
	**WL-RC**	0.443	0.015	−0.057	0.065	0.054	0	0	0.52
	**RP1**	−0.114	−0.005	0.184	−2.142	2.079	0	0	0
	**RP2**	0.003	0.000	−0.048	2.024	−4.207	2.228	0	0
	**RP3**	0.000	0.000	0.004	−0.497	1.729	−2.230	0.994	0
	**sum**	0	0	0	0	0.005	−0.002	0.994	1

**Table 2:** Thermodynamic properties of the model organisms. Location of the extremum of the bleach plus stimulated emission estimated from the SADS in Figure 5C, Figure 6C, Figure 7C and Figure 8C: $\lambda_{\max}$ in nm, ${\bar{\nu }}_{\max}$ in cm^−1^ and in k_B_T (1 k_B_T = 207 cm^−1^). ΔH relative to Bulk Chl *a* is in k_B_T. ΔG is taken from Figure 5A, Figure 6A, Figure 7A and Figure 8A.

A	*Chlamydomonas reinhardtii*	Ant1	Bulk Chl *a*	Red Chl *a*		WL-RC
	λ_max_ (nm)	675	682	689		690
	${\bar{\nu }}_{\max}$ (1/cm)	14,805	14,654	14,519		14,483
	${\bar{\nu }}_{\max}$ (kB.T)	72.7	72.0	71.3		71.1
	ΔH (kB.T)	0.7		−0.7		−0.8
	ΔG (kB.T)	4.1		1.3		1.3
	ΔH − ΔG = TΔS (kB.T)	−3.4		−2.0		−2.2
	“N”	2.7	78	10.4		8.7
						
B	*Synechocystis PCC6803*	Ant1	Bulk Chl *a*	Red Chl *a1*	Red Chl *a2*	WL-RC
	λ_max_ (nm)	683	685	701	709	693
	${\bar{\nu }}_{\max}$ (1/cm)	14,635	14,594	14,260	14,096	14,438
	${\bar{\nu }}_{\max}$ (kB.T)	71.9	71.7	70.0	69.2	70.9
	ΔH (kB.T)	0.2		−1.6	−2.4	−0.8
	ΔG (kB.T)	4.1		1.1	1.3	1.5
	ΔH – ΔG = TΔS (kB.T)	−3.9		−2.7	−3.8	−2.3
	“N”	1.6	78	5.3	1.8	8.1
						
C	*Thermosynechococcus elongatus*	Ant1	Bulk Chl *a*	Red Chl *a1*	Red Chl *a2*	WL-RC
	λ_max_ (nm)	681	686	707	723	693
	${\bar{\nu }}_{\max}$ (1/cm)	14,682	14,581	14,152	13,824	14,438
	${\bar{\nu }}_{\max}$ (kB.T)	72.1	71.6	69.5	67.9	70.9
	ΔH (kB.T)	0.5		−2.1	−3.7	−0.7
	ΔG (kB.T)	4.1		0.2	0.6	1.5
	ΔH – ΔG = TΔS (kB.T)	−3.6		−2.3	−4.3	−2.2
	“N”	2.1	78	7.9	1.0	8.6
						
D	*Spirulina platensis*	Ant1	Bulk Chl *a*	Red Chl *a1*	Red Chl *a2*	WL-RC
	λ_max_ (nm)	679	684	713	732	693
	${\bar{\nu }}_{\max}$ (1/cm)	14,722	14,630	14,031	13,670	14,438
	${\bar{\nu }}_{\max}$ (kB.T)	72.3	71.8	68.9	67.1	70.9
	ΔH (kB.T)	0.5		−2.9	−4.7	−0.9
	ΔG (kB.T)	4.1		0.1	−0.4	1.5
	ΔH – ΔG = TΔS (kB.T)	−3.7		−3.1	−4.3	−2.4
	“N”	2.0	78	3.6	1.0	6.8

ΔH − ΔG = TΔS (kB.T) and “N” computed via $N=N_{Bulk}\exp(\Delta S/k_{B})$.

## Data Availability

Representative figures of all data and fits are shown in Figures S2, S7, S10 and S13. The original raw data are available from the corresponding author upon reasonable request.

## Supplementary Materials

The supporting information can be downloaded at: https://www.mdpi.com/article/10.3390/ijms25074125/s1.

## Appendix A

### Model for the Observations

In transient-absorption spectroscopy, using pump and probe pulses of less than 100 fs FWHM, the evolution of the ground- and excited-state vibrational wave packets created by the short laser pulse is described with a superposition of damped oscillations. In addition, four-wave mixing contributes oscillations to the signals. The convolution with the IRF does not average out these oscillations, and they are clearly visible in the signals (Figure 3). The amplitude of a damped oscillation $\cos(\omega_{n}t)\exp(-\gamma_{n}t)$ as a function of the detection wavelength constitutes a damped-oscillation-associated spectrum ($DOAS_{n}(\lambda )$) with an accompanying wavelength-dependent phase $\varphi_{n}(\lambda )$ [45]. When the vibrational evolution can be considered independently from the electronic evolution (Born–Oppenheimer approximation), we arrive at a superposition of the electronic and vibrational contributions to the time-resolved spectrum (TRS):

$$TRS(t,\lambda )=\sum_{l=1}^{Nstates}c_{l}^{S}(t^{\prime },\theta )SADS_{l}(\lambda )+\sum_{n=1}^{Nosc}\cos(\omega_{n}t^{\prime }-\varphi_{n}(\lambda ))\exp(-\gamma_{n}t^{\prime })DOAS_{n}(\lambda )+\sum_{m=0}^{2}i^{\left(m\right)}(t)IRFAS_{m}(\lambda )+residual(t,\lambda )$$

where $t^{\prime }$ indicates that the actual model function still must consider the IRF by means of convolution, and $N_{states}$ electronically excited states are present in the system, with populations $c_{l}^{S}(t)$(superscript S stands for species) and species’ spectral properties, the $SADS_{l}(\lambda )$. The populations are determined by an unknown compartmental model [49] that depends upon the unknown kinetic parameter θ. In this *target* analysis, constraints on the *SADS* are needed to estimate all the θ parameters and the $SADS_{l}(\lambda )$. In the *global* analysis, we employ a sequential kinetic scheme with decreasing decay rates to arrive at the EADS, whereas a parallel kinetic scheme leads to the DADS. The third term describes the coherent artefact, with a weighted sum of the zeroth, first and second derivative of the IRF i(t) [46]. Finally, the residual represents the part of the data that is not described by the parameterized model.

For every wavelength, the matrix formula for this superposition model is given by

$$TRS=C^{S}(\theta ,\mu ,\Delta )\cdot SADS+\mathrm{Cos}(\omega ,\gamma ,\mu ,\Delta )\cdot A+\mathrm{Sin}(\omega ,\gamma ,\mu ,\Delta )\cdot B+IRF(\mu ,\Delta \prime )\cdot IRFAS$$

where the matrix $C^{S}$ consists of columns $c_{l}^{S}(t)$. A Gaussian-shaped IRF is used, with parameters μ for the time of the IRF maximum and Δ for the FWHM of the IRF. The matrices Cos(ω,γ,μ,Δ) and Sin(ω,γ,μ,Δ) contain the damped oscillations, and the matrices A and B comprise their amplitudes. To limit the number of free parameters, we assume wavelength independence of the eigenfrequency $\omega_{n}$ and of the damping rate $\gamma_{n}$.

The final term, which describes the coherent artefact, contains the matrix IRF(μ,Δ′) with as columns the $i^{\left(m\right)}(t)$. The SADS and IRFAS and also the amplitudes A and B are unconstrained conditionally linear parameters, which can be implicitly solved (per wavelength) using the variable-projection algorithm [50,51].

For each experiment, the matrix of the residuals is analyzed with the help of a singular value decomposition [17,52]:

$$residual(t,\lambda )=\sum_{i=1}^{m}lsv_{i}(t)\cdot s_{i}\cdot rsv_{i}(\lambda )$$

Here, the time and wavelength indices run from 1 until nt and nλ and m=min(nt,nλ), respectively. Typically, after a satisfactory fit, the first singular value *s_1_* dominates, and the first left and right singular vectors (lsv and rsv) reveal the remaining dominant patterns or trends in the residuals, which can be attributed to fluctuations of the probe white light that was generated using a single filament (Figures S3, S8, S11 and S14).
