# Supplementary material for: Energy Transfer and Radical-Pair Dynamics in Photosystem I with Different Red Chlorophyll a Pigments
Source: Int J Mol Sci. 2024 Apr 8;25(7):4125. doi: 10.3390/ijms25074125 (PMC11012434; doi:10.3390/ijms25074125)
Supplement: Supplementary file 1 [file ijms-25-04125-s001.zip › ijms-2935383-supplementary.pdf]

Supplementary Materials to

Energy transfer and radical pair dynamics in photosystem I with different red Chlorophyll *a*

Ivo H.M. van Stokkum[1*,†], Marc G. Müller[2,†], Alfred R. Holzwarth[1,2]

[1]Department of Physics and Astronomy and LaserLaB, Faculty of Science, Vrije Universiteit Amsterdam, De Boelelaan 1081, 1081 HV, Amsterdam, The Netherlands

[2]Max-Planck-Institut für chemische Energiekonversion, D-45470 Mülheim a.d. Ruhr, Germany

*Correspondence: i.h.m.van.stokkum@vu.nl

†contributed equally

Sub-ps analysis and fit quality of the Target Analysis of the transient absorption dynamics in reduced PSI of *Chlamydomonas reinhardtii*

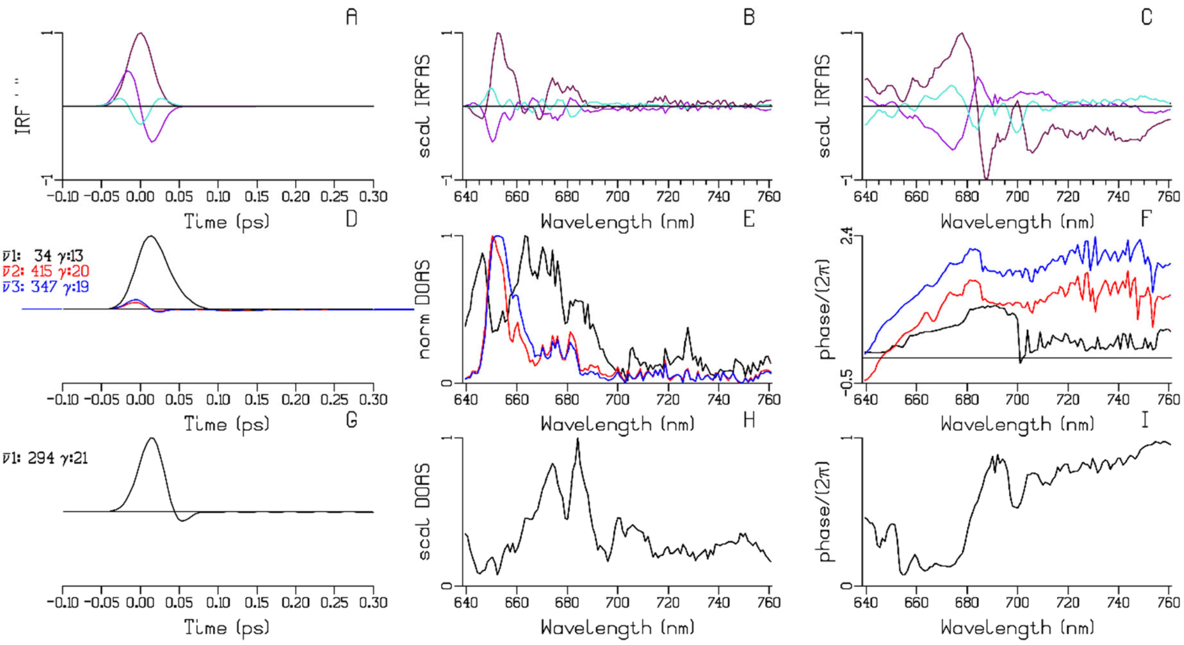

Figure S1. Overview of the analysis of the sub-ps dynamics in the reduced PSI complex of *Chlamydomonas reinhardtii* (related to Figure 5). (A) zeroth, first and second derivative of the IRF with 670 nm excitation (maroon, purple and turquoise, respectively). (B) scaled IRFAS. Scaling of the IRFAS is such that the product of the IRFAS and the IRF derivative is the contribution to the fit. Thus, the maroon IRFAS has the largest contribution to the fit. (C) scaled IRFAS with 700 nm excitation. In addition, damped oscillations are present [45] (D-F) with 670 nm excitation (G-I) with 700 nm excitation. (D, G) Cosine oscillations with frequencies $\bar{\nu}n$ (in cm^{-1}) (where *n* is the DOAS number) and damping rates γ (in ps^{-1}) written in the legend at the left, using the appropriate color. (E,H) Estimated DOAS (with number and color indicated in the legend at the far left), scaled for comparison. Scaling of the DOAS in H is such that the product of the DOAS and the damped oscillation is the contribution to the fit. In E the normalized DOAS are depicted (F,I) Estimated phase profiles of the DOAS (with number and color indicated in the legend at the far left).

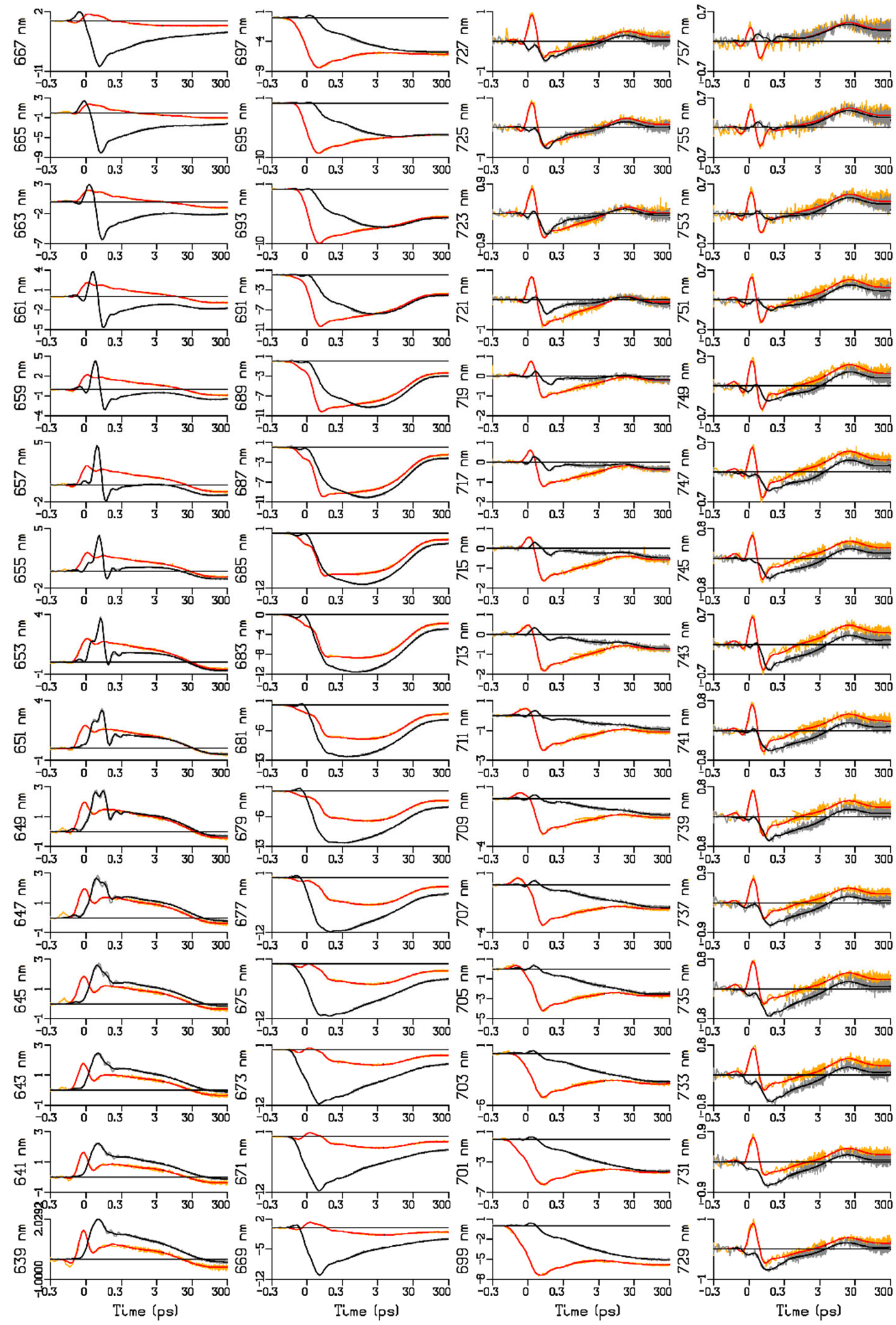

Figure S2. Transient absorption (in units of mOD) of *Chlamydomonas reinhardtii* WL-PSI in the reduced form at 60 selected wavelengths (indicated in the ordinate label) (related to Figure 3). Key: 670 nm excitation (grey), 700 nm excitation (orange). Black and red lines indicate the target analysis fit. Note that the time axis is linear until 0.3 ps and logarithmic thereafter. Note also that each panel is scaled to its maximum. The overall rms error of the fit was 0.056 mOD.

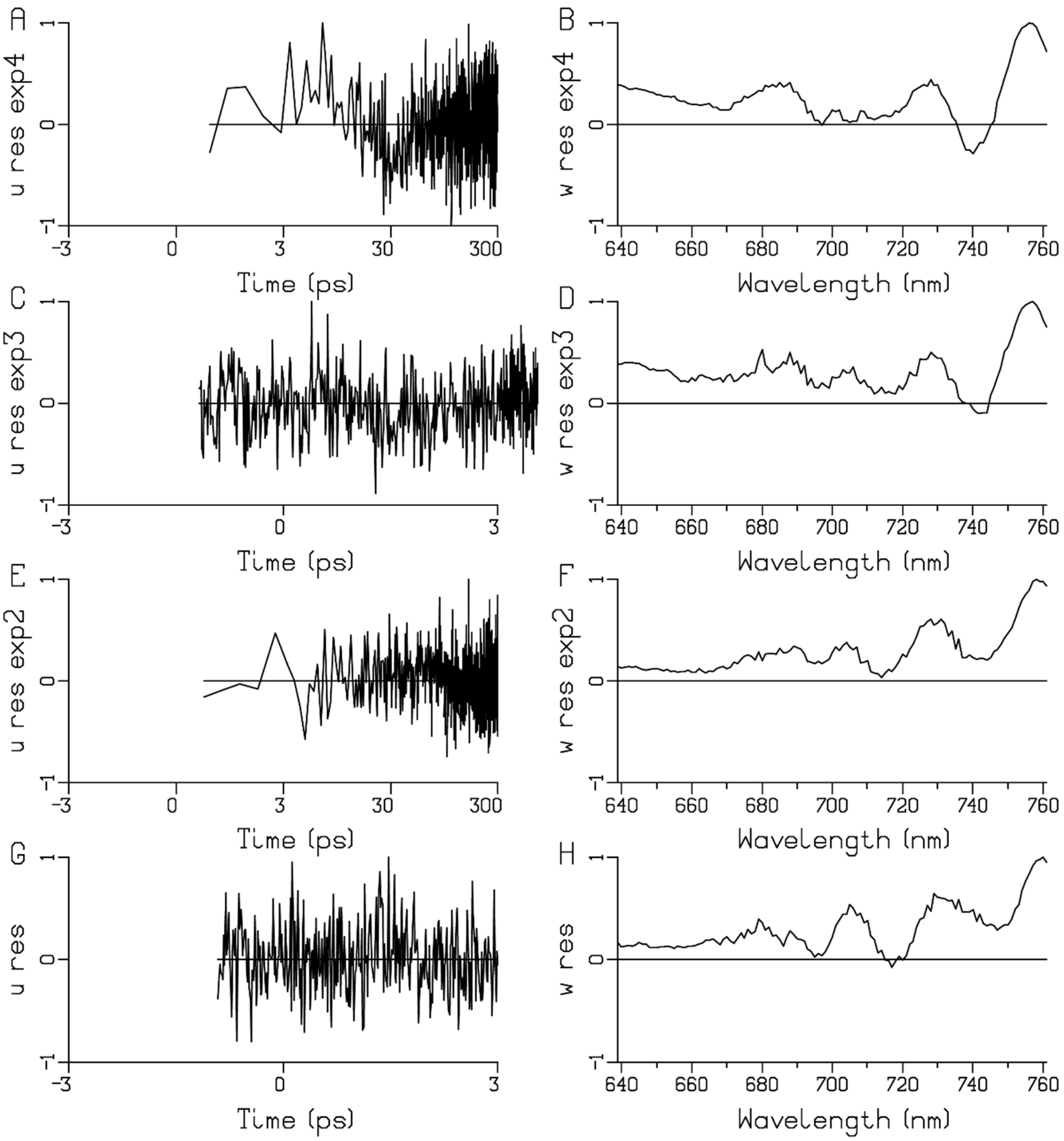

Figure S3. First left (A,C,E,G) and right (B,D,F,H) singular vectors resulting from the singular value decomposition (SVD) of the residual matrix of WL-PSI of WT *Chlamydomonas reinhardtii* resulting from the *target* analysis (related to Figure 3). Note that all first left singular vector panels (A,C,E,G) show no trends at all. The pattern in the right singular vectors can be interpreted as fluctuations of the probe white light that was generated using a single filament, and thus is expected to increase towards 800 nm. Key A,B: WL 700 nm excitation TimeRange(TR)2; C,D: WL 700 nm excitation TR1; E,F: WL 670 nm excitation TR2; G,H: WL 670 nm excitation TR1. Note that the time axis in (A,C,E,G) is linear until 3 ps and logarithmic thereafter.

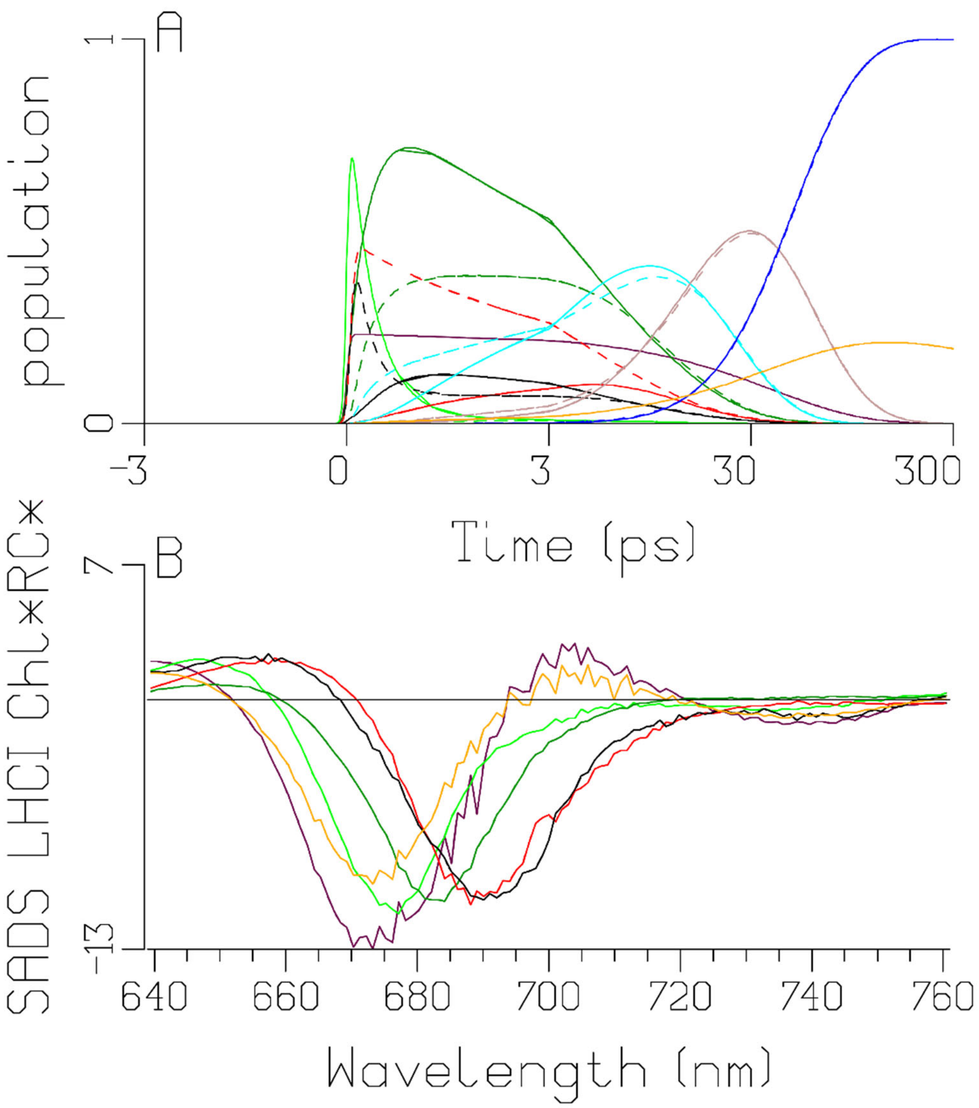

Figure S4. Populations (A) and SADS (B, in mOD). Line type key: 670 nm excitation (solid), 700 nm excitation (dashed). Color key: Ant1 (light green), Bulk Chl *a* (dark green), Red Chl *a* (red), WL-RC (black), RP1 (cyan), RP2 (brown), RP3 (blue), and LHCI (maroon and orange). Note that the time axis in (A) is linear until 3 ps and logarithmic thereafter. Only with 670 nm excitation about 20% of the photons is absorbed by an LHCI contamination, that equilibrates in 39 ps and subsequently decays in 1.44 ns. The LHCI SADS are at higher energy than Ant1.

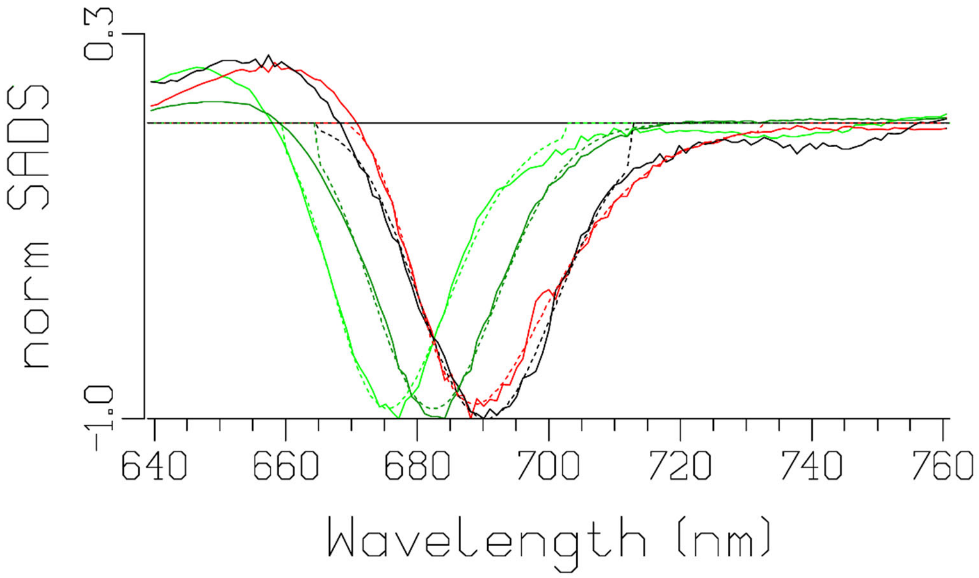

Figure S5. Normalized excited state SADS of *Chlamydomonas reinhardtii* (A) with superimposed spectral fits (dotted lines) using skewed Gaussian shapes (B) (related to Figure 5). Key: Ant1 (light green), Bulk Chl *a* (dark green), Red Chl *a* (red), WL-RC (black). Note that a single skewed Gaussian shape cannot well describe the SE of the vibrational tails nor the ESA.

Sub-ps analysis and fit quality of the Target Analysis of the transient absorption dynamics in reduced PSI of *Synechocystis* PCC6803

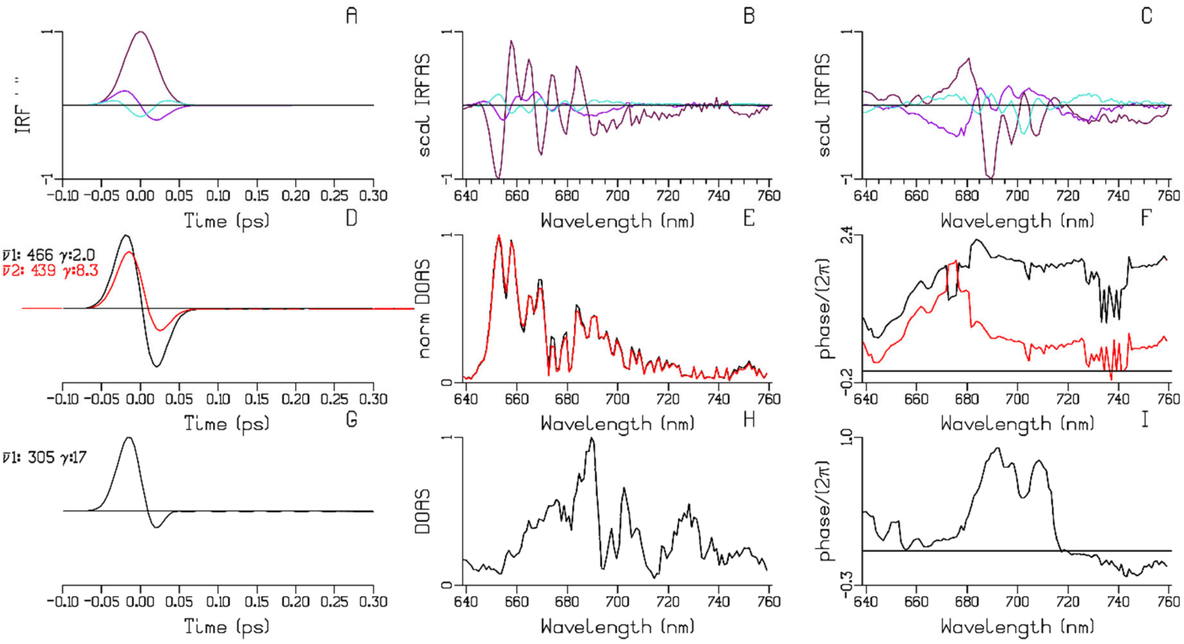

Figure S6. Overview of the analysis of the sub-ps dynamics in the reduced PSI complex of *Synechocystis* PCC6803 (related to Figure 6). (A) zeroth, first and second derivative of the IRF with 670 nm excitation (maroon, purple and turquoise, respectively). (B) scaled IRFAS. Scaling of the IRFAS is such that the product of the IRFAS and the IRF derivative is the contribution to the fit. Thus, the maroon IRFAS has the largest contribution to the fit. (C) scaled IRFAS with 700 nm excitation. In addition, damped oscillations are present [45] (D-F) with 670 nm excitation (G-I) with 700 nm excitation. (D, G) Cosine oscillations with frequencies $\bar{\nu}n$ (in cm^{-1}) (where *n* is the DOAS number) and damping rates γ (in ps^{-1}) written in the legend at the left, using the appropriate color. (E,H) Estimated DOAS (with number and color indicated in the legend at the far left), scaled for comparison. Scaling of the DOAS in H is such that the product of the DOAS and the damped oscillation is the contribution to the fit. In E the normalized DOAS are depicted (F,I) Estimated phase profiles of the DOAS (with number and color indicated in the legend at the far left).

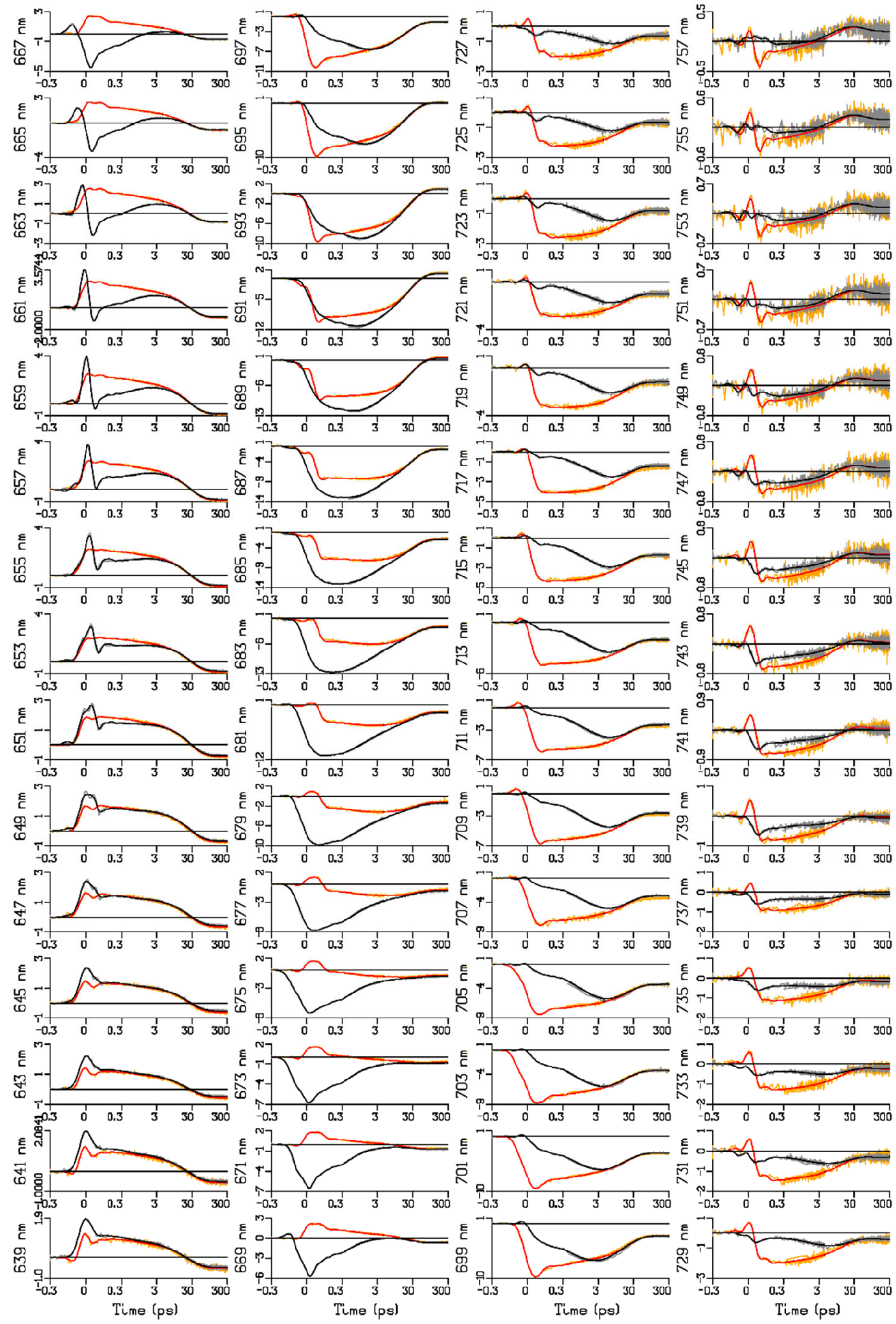

Figure S7. Transient absorption (in units of mOD) of *Synechocystis* PCC6803 WL-PSI in the reduced form at 60 selected wavelengths (indicated in the ordinate label) (related to Figure 6). Key: 670 nm excitation (grey), 700 nm excitation (orange). Black and red lines indicate the target analysis fit. Note that the time axis is linear until 0.3 ps and logarithmic thereafter. Note also that each panel is scaled to its maximum. The overall rms error of the fit was 0.067 mOD.

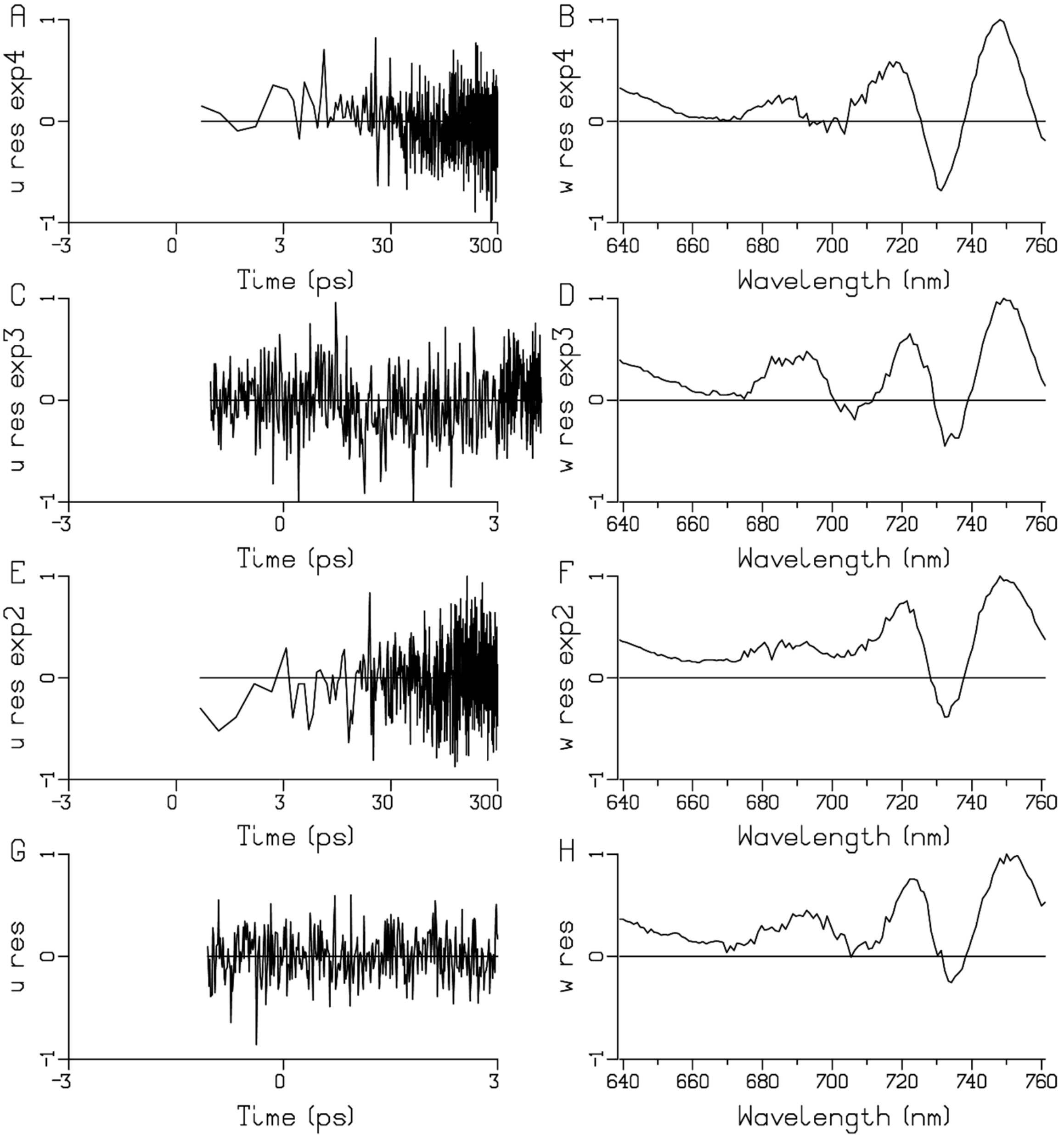

Figure S8. First left (A,C,E,G) and right (B,D,F,H) singular vectors resulting from the singular value decomposition (SVD) of the residual matrix of WL-PSI of WT *Synechocystis* PCC6803 resulting from the target analysis (related to Figure 6). Note that all first left singular vector panels (A,C,E,G) show no trends at all. The pattern in the right singular vectors can be interpreted as fluctuations of the probe white light that was generated using a single filament, and thus is expected to increase towards 800 nm. Key A,B: WL 700 nm excitation TimeRange(TR)2; C,D: WL 700 nm excitation TR1; E,F: WL 670 nm excitation TR2; G,H: WL 670 nm excitation TR1. Note that the time axis in (A,C,E,G) is linear until 3 ps and logarithmic thereafter.

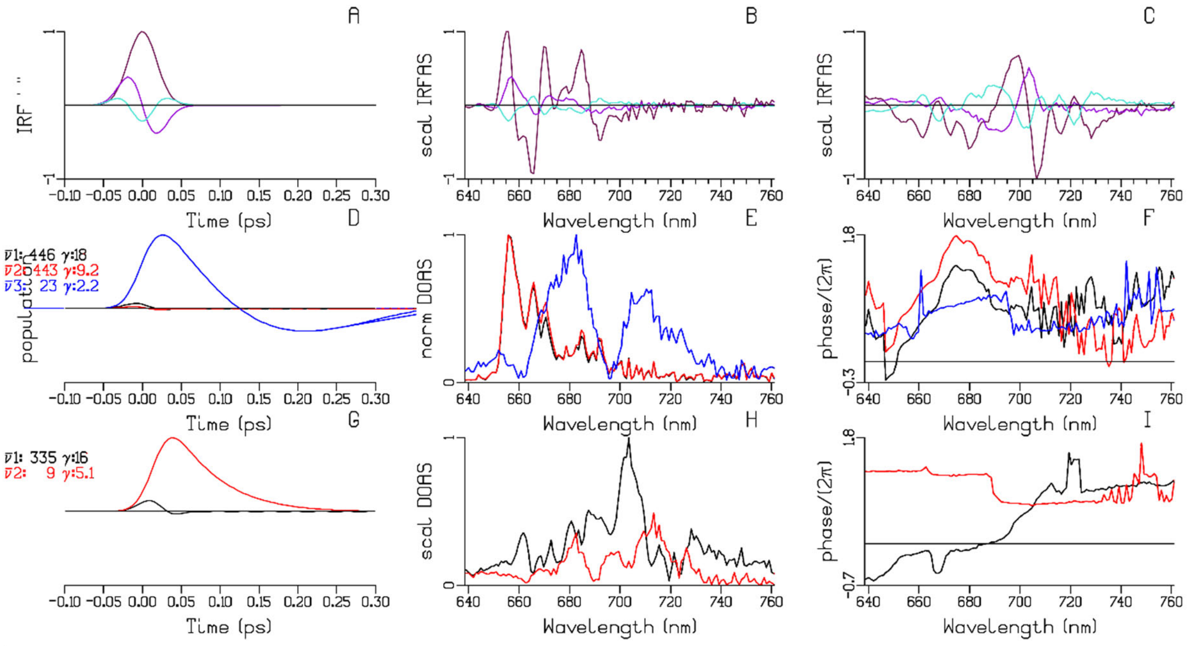

Figure S9. Overview of the analysis of the sub-ps dynamics in the reduced PSI complex of *Thermosynechococcus elongatus* (related to Figure 7). (A) zeroth, first and second derivative of the IRF with 670 nm excitation (maroon, purple and turquoise, respectively). (B) scaled IRFAS. Scaling of the IRFAS is such that the product of the IRFAS and the IRF derivative is the contribution to the fit. Thus, the maroon IRFAS has the largest contribution to the fit. (C) scaled IRFAS with 720 nm excitation. In addition, damped oscillations are present [45] (D-F) with 670 nm excitation (G-I) with 700 nm excitation. (D, G) Cosine oscillations with frequencies $\overline{\nu n}$ (in cm^{-1}) (where *n* is the DOAS number) and damping rates γ (in ps^{-1}) written in the legend at the left, using the appropriate color. (E,H) Estimated DOAS (with number and color indicated in the legend at the far left), scaled for comparison. Scaling of the DOAS in H is such that the product of the DOAS and the damped oscillation is the contribution to the fit. In E the normalized DOAS are depicted (F,I) Estimated phase profiles of the DOAS (with number and color indicated in the legend at the far left).

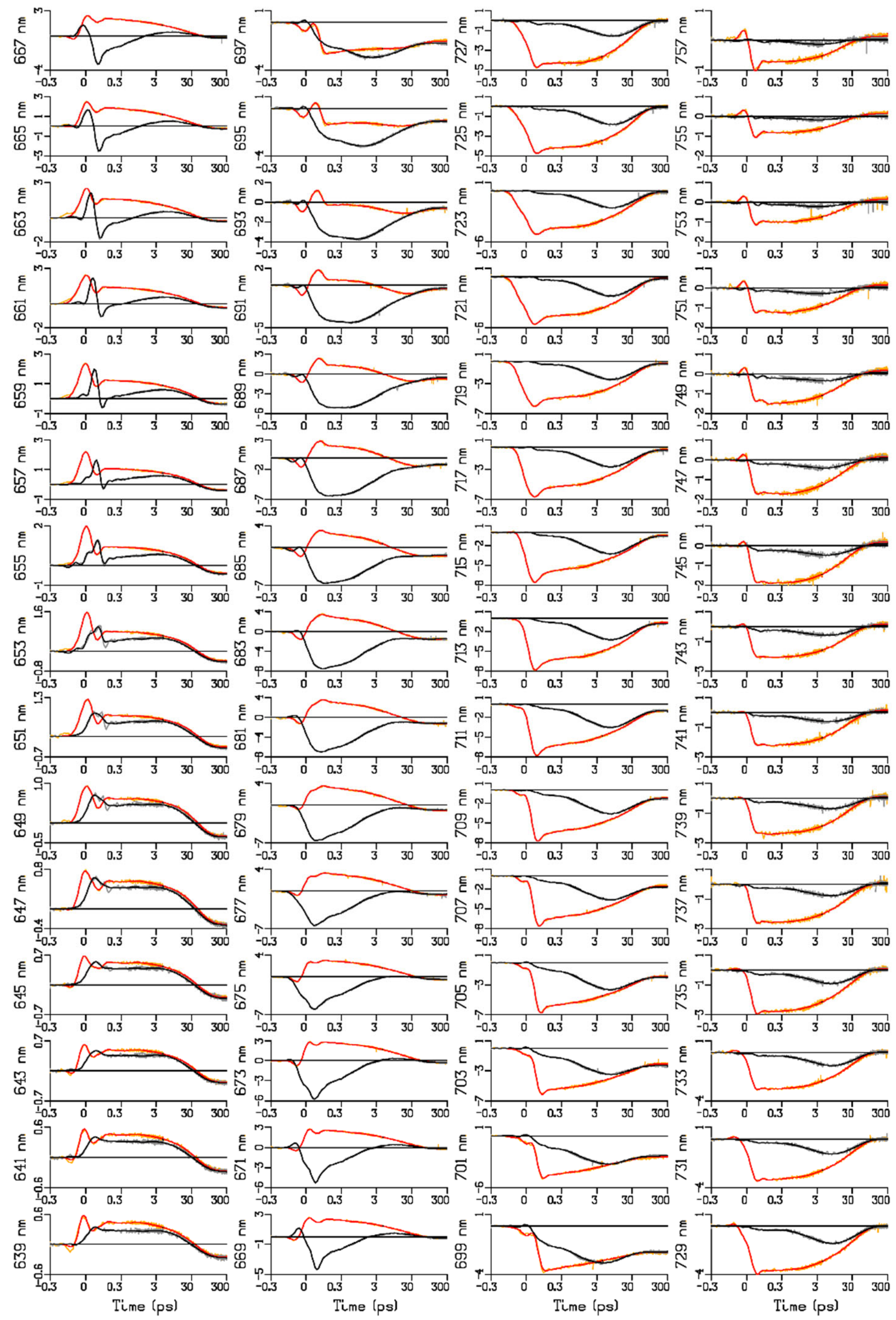

Figure S10. Transient absorption (in units of mOD) of *Thermosynechococcus elongatus* WL-PSI in the reduced form at 60 selected wavelengths (indicated in the ordinate label) (related to Figure 7). Key: 670 nm excitation (grey), 720 nm excitation (orange). Black and red lines indicate the target analysis fit. Note that the time axis is linear until 0.3 ps and logarithmic thereafter. Note also that each panel is scaled to its maximum. The overall rms error of the fit was 0.054 mOD.

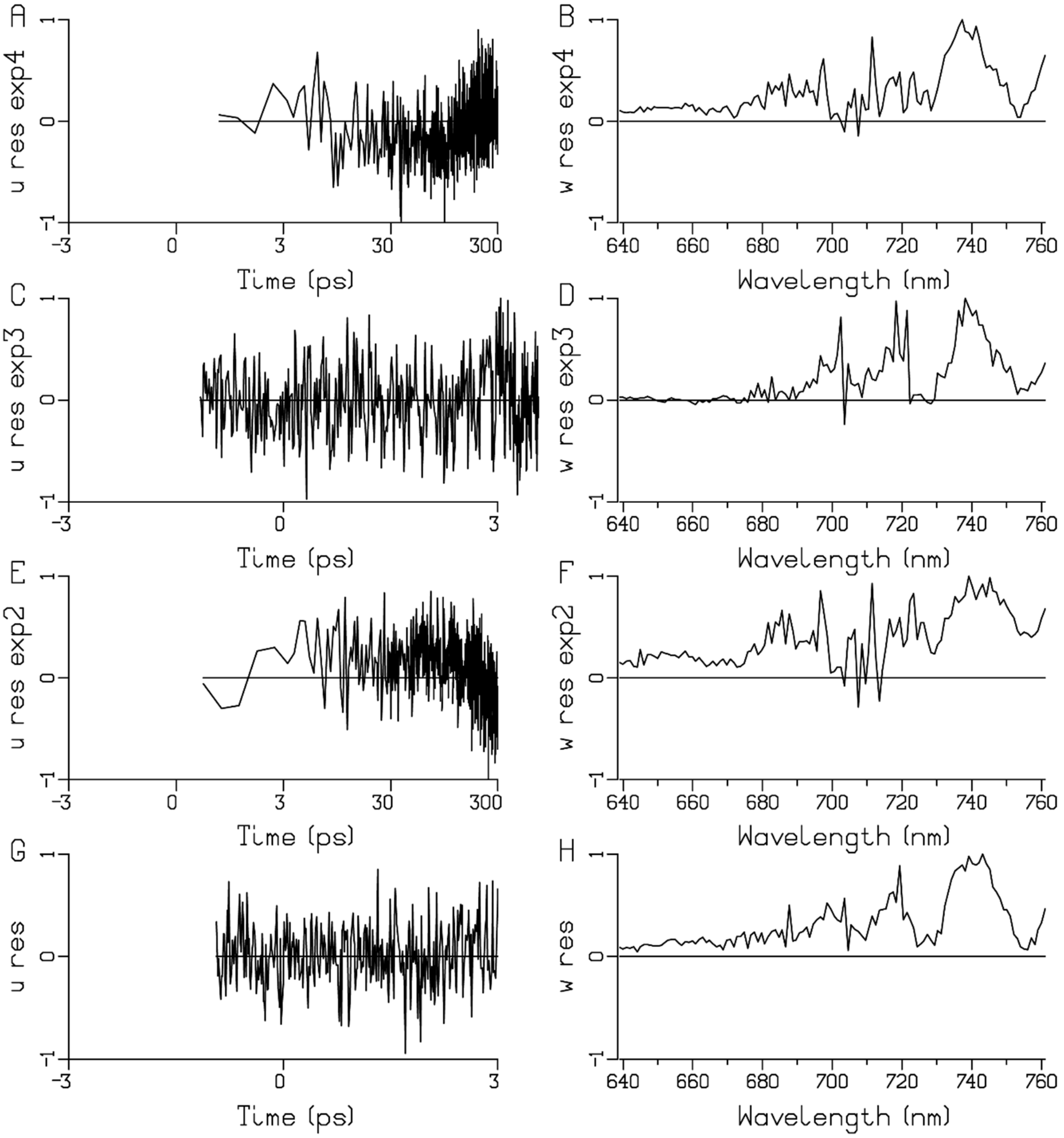

Figure S11. First left (A,C,E,G) and right (B,D,F,H) singular vectors resulting from the singular value decomposition (SVD) of the residual matrix of WL-PSI of WT *Thermosynechococcus elongatus* resulting from the *target* analysis (related to Figure 7). Note that all first left singular vector panels (A,C,E,G) show no trends at all. The pattern in the right singular vectors can be interpreted as fluctuations of the probe white light that was generated using a single filament, and thus is expected to increase towards 800 nm. Key A,B: WL 720 nm excitation TimeRange(TR)2; C,D: WL 720 nm excitation TR1; E,F: WL 670 nm excitation TR2; G,H: WL 670 nm excitation TR1. Note that the time axis in (A,C,E,G) is linear until 3 ps and logarithmic thereafter.

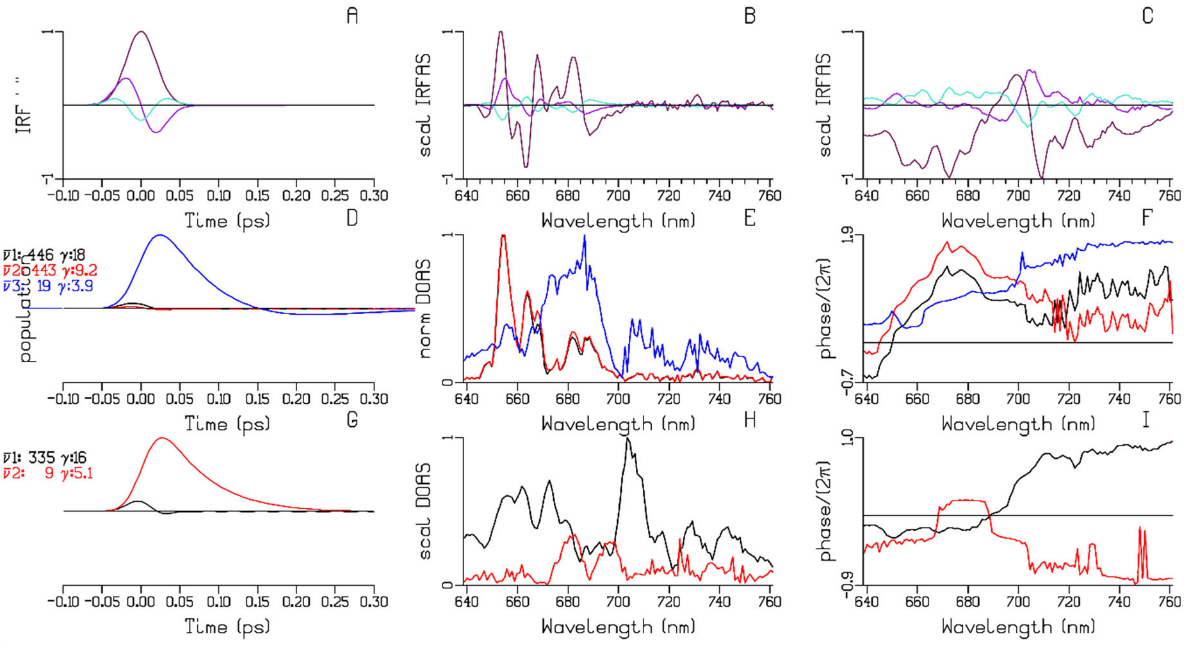

Figure S12. Overview of the analysis of the sub-ps dynamics in the reduced PSI complex of *Spirulina platensis* (related to Figure 8). (A) zeroth, first and second derivative of the IRF with 670 nm excitation (maroon, purple and turquoise, respectively). (B) scaled IRFAS. Scaling of the IRFAS is such that the product of the IRFAS and the IRF derivative is the contribution to the fit. Thus, the maroon IRFAS has the largest contribution to the fit. (C) scaled IRFAS with 720 nm excitation. In addition, damped oscillations are present [45] (D-F) with 670 nm excitation (G-I) with 720 nm excitation. (D, G) Cosine oscillations with frequencies $\overline{\nu}n$ (in cm^{-1}) (where *n* is the DOAS number) and damping rates γ (in ps^{-1}) written in the legend at the left, using the appropriate color. (E,H) Estimated DOAS (with number and color indicated in the legend at the far left), scaled for comparison. Scaling of the DOAS in H is such that the product of the DOAS and the damped oscillation is the contribution to the fit. In E the normalized DOAS are depicted (F,I) Estimated phase profiles of the DOAS (with number and color indicated in the legend at the far left).

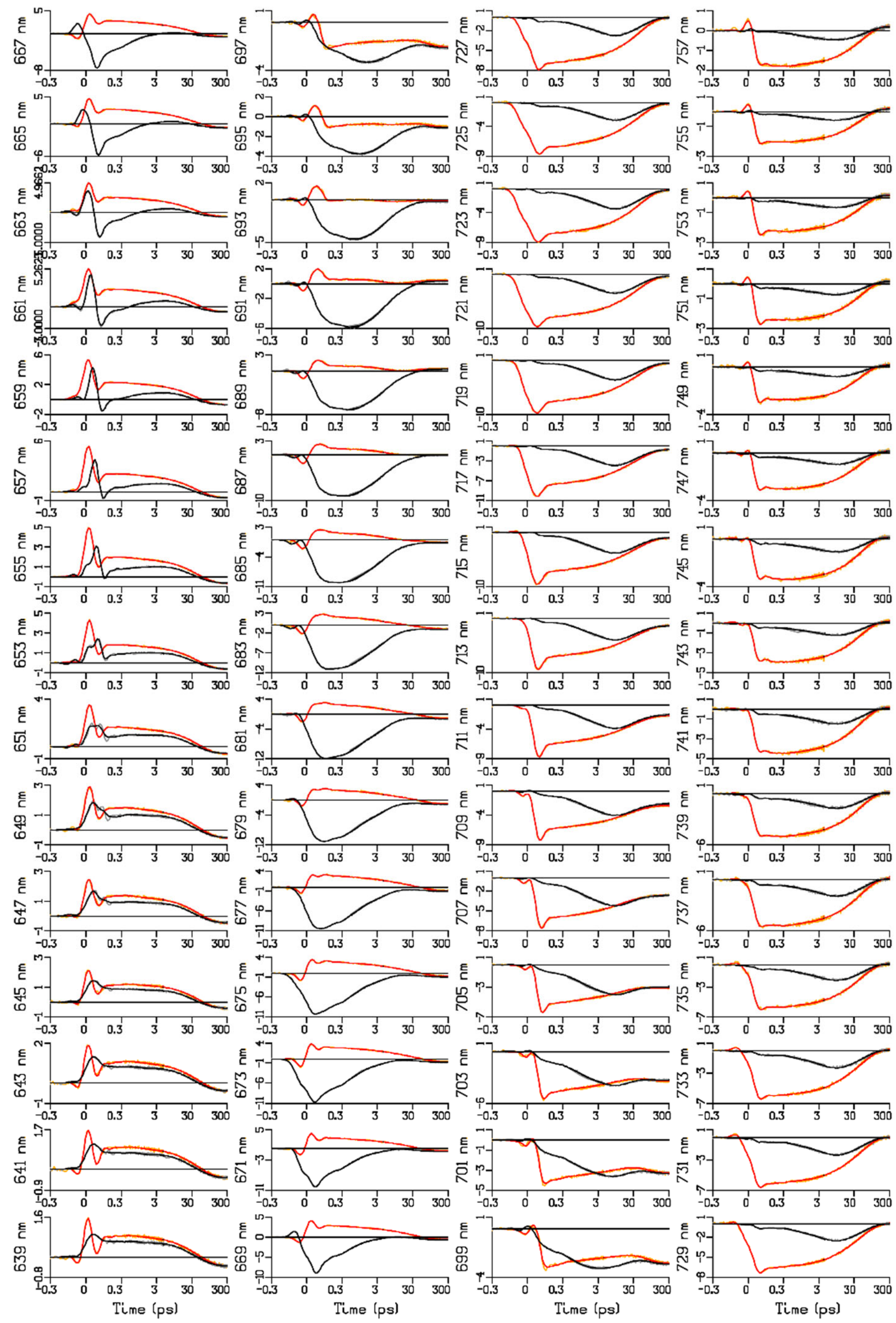

Figure S13. Transient absorption (in units of mOD) of *Spirulina platensis* WL-PSI in the reduced form at 60 selected wavelengths (indicated in the ordinate label) (related to Figure 8). Key: 670 nm excitation (grey), 720 nm excitation (orange). Black and red lines indicate the target analysis fit. Note that the time axis is linear until 0.3 ps and logarithmic thereafter. Note also that each panel is scaled to its maximum. The overall rms error of the fit was 0.050 mOD.

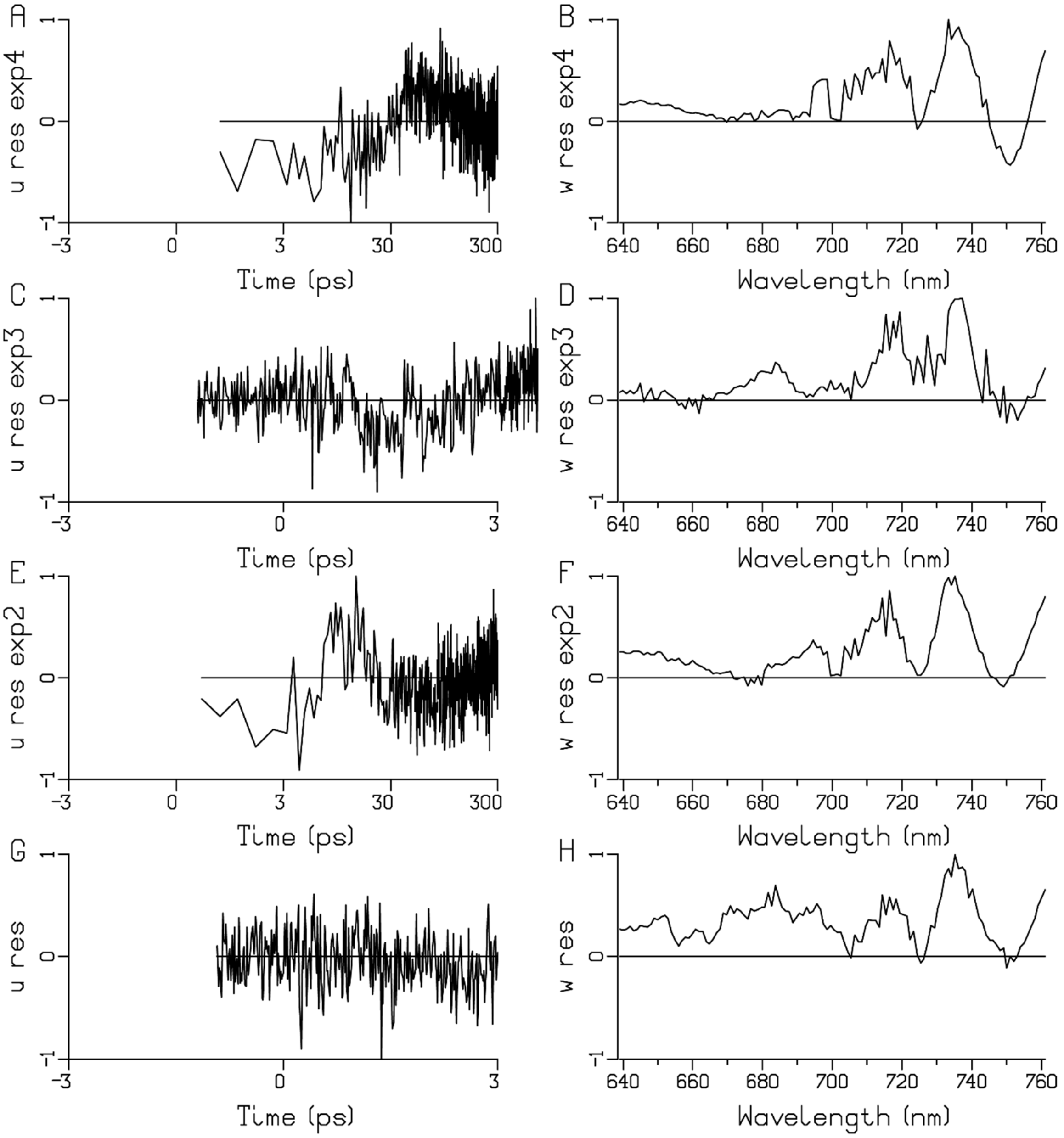

Figure S14. First left (A,C,E,G) and right (B,D,F,H) singular vectors resulting from the singular value decomposition (SVD) of the residual matrix of WL-PSI of WT *Spirulina platensis* resulting from the *target* analysis (related to Figure 8). Note that all first left singular vector panels (A,C,E,G) show no trends at all. The pattern in the right singular vectors can be interpreted as fluctuations of the probe white light that was generated using a single filament, and thus is expected to increase towards 800 nm. Key A,B: WL 720 nm excitation TimeRange(TR)2; C,D: WL 720 nm excitation TR1; E,F: WL 670 nm excitation TR2; G,H: WL 670 nm excitation TR1. Note that the time axis in (A,C,E,G) is linear until 3 ps and logarithmic thereafter.

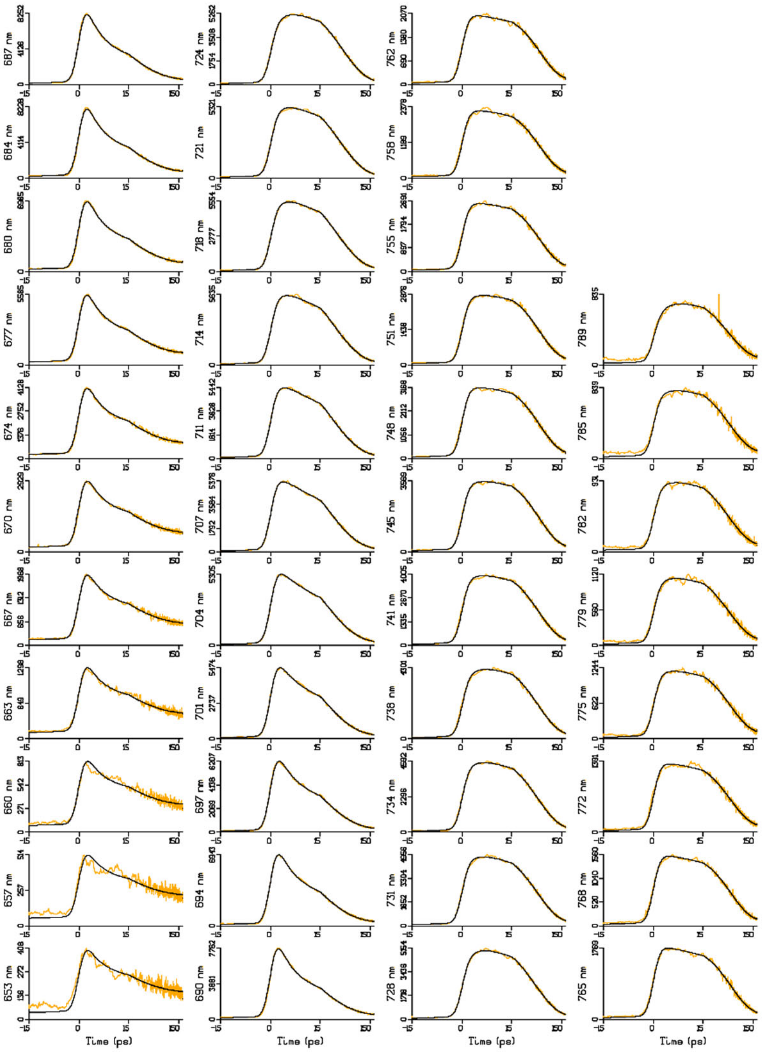

Figure S15. Emission of *Spirulina platensis* WL-PSI at 41 wavelengths (indicated in the ordinate label) (related to Figure 9). Key: data (orange) and target analysis fit (black). Note that the time axis is linear until 15 ps and logarithmic thereafter. Note also that each panel is scaled to its maximum. The overall rms error of the fit was 47.
